# Sleep deprivation leads to non-adaptive alterations in sleep microarchitecture and amyloid-β accumulation in a murine Alzheimer model

**DOI:** 10.1016/j.celrep.2024.114977

**Published:** 2024-11-15

**Authors:** Neža Cankar, Natalie Beschorner, Anastasia Tsopanidou, Filippa L. Qvist, Ana R. Colaço, Mie Andersen, Celia Kjaerby, Christine Delle, Marius Lambert, Filip Mundt, Pia Weikop, Mathias Jucker, Matthias Mann, Niels Henning Skotte, Maiken Nedergaard

**Affiliations:** 1 Center for Translational Neuromedicine, Faculty of Medical and Health Sciences, University of Copenhagen, Blegdamsvej 3B, 2200 Copenhagen N, Denmark; 2 Center for Translational Neuromedicine, University of Rochester Medical School, Elmwood Avenue 601, Rochester, NY 14642, USA; 3 NNF Center for Protein Research, Faculty of Health Sciences, University of Copenhagen, Copenhagen, Denmark; 4 Department of Drug Design and Pharmacology, University of Copenhagen, Copenhagen, Denmark; 5 Department of Cellular Neurology, Hertie Institute for Clinical Brain Research, University of Tübingen, Tübingen, Germany; 6 Department for Proteomics and Signal Transduction, Max-Planck Institute for Biochemistry, Am Klopferspitz 18, 82152 Martinsried, Germany; 7 Proteomics Research Infrastructure, Novo Nordisk Foundation Center for Protein Research, Faculty of Health and Medical Sciences, University of Copenhagen, Blegdamsvej 3B, 2200 Copenhagen N, Denmark; 8 These authors contributed equally; 9 Lead contact

## Abstract

Impaired sleep is a common aspect of aging and often precedes the onset of Alzheimer’s disease. Here, we compare the effects of sleep deprivation in young wild-type mice and their APP/PS1 littermates, a murine model of Alzheimer’s disease. After 7 h of sleep deprivation, both genotypes exhibit an increase in EEG slow-wave activity. However, only the wild-type mice demonstrate an increase in the power of infraslow norepinephrine oscillations, which are characteristic of healthy non-rapid eye movement sleep. Notably, the APP/PS1 mice fail to enhance norepinephrine oscillations 24 h after sleep deprivation, coinciding with an accumulation of cerebral amyloid-β protein. Proteome analysis of cerebrospinal fluid and extracellular fluid further supports these findings by showing altered protein clearance in APP/PS1 mice. We propose that the suppression of infraslow norepinephrine oscillations following sleep deprivation contributes to increased vulnerability to sleep loss and heightens the risk of developing amyloid pathology in early stages of Alzheimer’s disease.

## In brief

Cankar et al. report that sleep loss suppresses NREM norepinephrine oscillations in a murine model of Alzheimer’s disease along with a parallel buildup of amyloid-β. Thus, overexpression of amyloid-β inhibits recovery sleep and thereby slows glymphatic clearance after sleep deprivation. Norepinephrine oscillations persisted in wild-type mice after sleep deprivation.

## Graphical Abstract

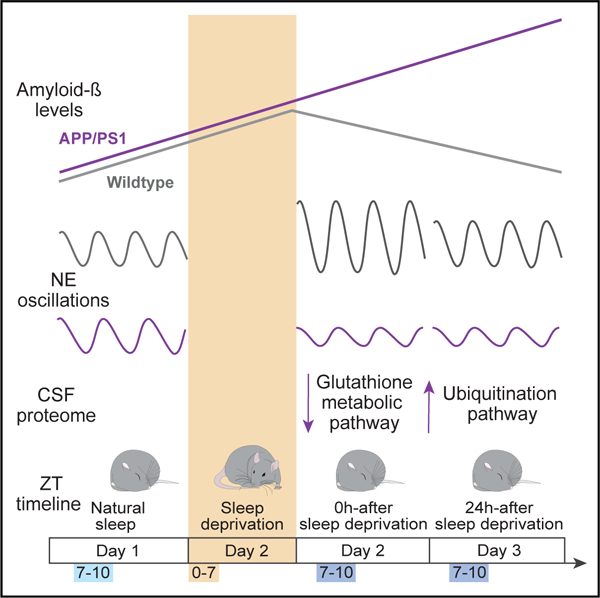

## INTRODUCTION

Poor sleep quality is common among the elderly and may precede symptomatic onset of neurodegenerative disorders associated with protein aggregation, such as Alzheimer’s disease (AD).^1,2^ Sleep disruptions often manifest prior to clinical diagnosis of AD and may even predict its onset.^3,4^ Studies of otherwise healthy night-shift workers have reported an increased predisposition to develop cognitive impairments and higher incidence of dementia.^5,6^ Thus, mechanistic insight into how impaired sleep contributes to initiation and progression of protein aggregation and neurodegeneration is needed.

The glymphatic system plays a pivotal role in regulating the central nervous system (CNS) proteostasis during sleep.^7,8^ As a brain-wide clearance system, it facilitates removal of metabolic waste and toxic extracellular proteins, such as amyloid-β (Aβ), tau, and α-synuclein (Snca).^8–10^ Glymphatic inflow of cerebrospinal fluid (CSF) along the periarterial spaces is driven by pulsatility of the vessel wall.^11,12^ The high expression of the aquaporin 4 (AQP4) water channel in the vascular endfeet of astrocytes facilitates parenchymal influx of CSF.^11^ The extracellular fluid and proteins exit from the parenchyma via perivenous spaces and along cranial and spinal nerves. Ultimately, the fluid enters meningeal lymphatic vessels and drains into the cervical lymph nodes.^13,14^ Glymphatic clearance has received considerable interest in the field of neurodegenerative diseases due to its importance in clearing of brain proteins. Glymphatic activity peaks during non-rapid eye movement (NREM) sleep and is suppressed during wakefulness.^7^ Thus, sleep disturbances are predicted to reduce protein clearance. Indeed, a positron emission tomography (PET) study showed that a single night of sleep deprivation increased Aβ accumulation in the hippocampus and thalamus of healthy volunteers.^15^ In mice, prolonged wakefulness elevated levels of Aβ and tau both in the extracellular fluid and in the CSF.^10,16^ During recovery sleep, which is a compensatory phase following sleep deprivation wherein the brain restores its functions, NREM slow-wave activity (SWA) increases.^17,18^ The SWA magnitude correlates well with the duration of pre-existing sleep debt and promotes glymphatic fluid flow.^7,17–19^ Although the acute effects of sleep deprivation on cerebral Aβ levels are well documented, the effect of subsequent recovery sleep on brain Aβ clearance remains unexplored in AD brain.

Norepinephrine (NE) plays a crucial role in regulating several essential functions within the CNS, including arousal state, glymphatic flow, and the orchestration of the temporal microarchitecture of NREM sleep.^20–22^ Infraslow spontaneous NE oscillations (~30 s) during NREM correlated positively with memory performance, while suppression of NE oscillations compromised cognitive function in mice.^20^ NE is a potent vasoconstrictor that regulates vascular tone,^23^ and modeling studies have predicted that the NE oscillations drive perivascular glymphatic flow.^21^ NE signaling is compromised early in AD, and locus coeruleus degeneration may contribute to disease progression.^24,25^ While the globally high extracellular levels of NE during wakefulness directly inhibit glymphatic activity,^7^ less is known about how infraslow NE oscillatory dynamics change during disease. To our knowledge, the role of NE oscillations after sleep deprivation has not yet been determined.

Here, we investigated the effect of sleep loss on the clearance of brain Aβ in young pre-depositing (3–4 months of age) APP/PS1 mice and their non-transgenic wild-type (WT) littermates, while comparing NE oscillatory dynamic changes in NREM sleep 24 h after sleep deprivation. Our findings revealed that abnormal sleep-wake cycles, coupled with impaired glymphatic efflux, preceded the formation of amyloid plaques. Notably, young APP/PS1 mice exhibited a failure to increase NE oscillatory power 24 h after sleep deprivation, which was accompanied by elevated levels of the cerebral Aβ_42_ isoform. This accumulation of Aβ following sleep deprivation was further corroborated by changes in the CSF proteome, indicating a disruption in intracellular protein processing. In addition, by combining microdialysis with unbiased proteomics, we introduce an approach for characterizing the regional brain extracellular fluid proteome.

## RESULTS

### Young APP/PS1 mice show abnormal sleep architecture prior to Aβ plaque deposition

**Figure 1.**
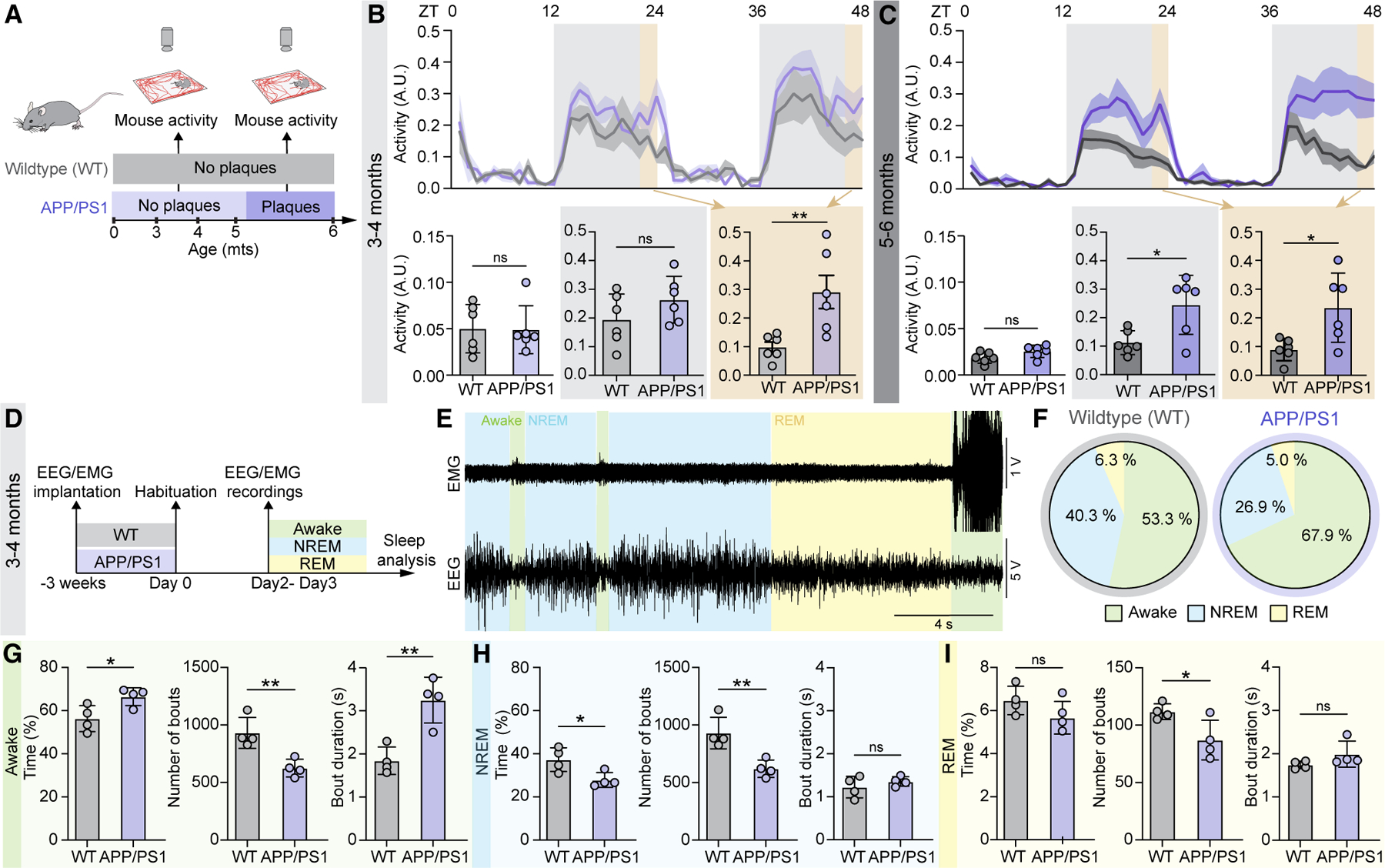
Sleep is shorter in young APP/PS1 mice (A) Scheme illustrating the mouse activity experimental setup. (B and C) Activity tracking of WT and APP/PS1 mice during the active phase (ZT 12–24 and ZT 36–48) and inactive phase (ZT 0–12 and ZT 24–36), indicated in arbitrary units (A.U.) at (B) 3–4 months and (C) 5–6 months of age. Traces indicate mean ± SEM. Mean activity within each phase is shown at the bottom; *p = 0.0158. Orange bar indicates 1-h time window at the end of active phase; **p = 0.0099, *p = 0.0180, *n* = 6, unpaired t test. (D) Schematic of EEG recording experiments. (E) Representative EEG/EMG trace for one APP/PS1 mouse. (F) Pie chart showing total percentage of time spent awake, in NREM sleep, and in REM sleep across a 48-h duration in the 3–4 months group, *n* = 4. (G–I) Total percentage of time, duration, and bout assessment of (G) wakefulness, *p = 0.031, **p = 0.0081, and **p = 0.0048; (H) NREM sleep, *p = 0.027, **p = 0.0070, and ns = 0.039; and (I) REM sleep, ns = 0.145, *p = 0.00182, and ns = 0.135, unpaired t test, *n* = 4. All bar graphs show ±SD.

We first assessed whether changes in sleep-wake rhythms appear prior to Aβ aggregation in APP/PS1 using 48-h activity tracking (Figure 1A). Aβ plaque deposition in APP/PS1 mice first emerges at around 6 months of age.^26^ The mean activities during the entire active (dark-cycle) and inactive (light-cycle) phases did not differ between APP/PS1 and WT mice at 3–4 months of age (Figure 1B, active, WT 0.049 ± 0.026 a.u., APP/PS1 0.048 ± 0.026 a.u., and inactive, WT 0.19 ± 0.089 a.u., APP/PS1 0.26 ± 0.081 a.u.). Patients with AD often suffer from ‘‘sundown syndrome,’’ characterized by anxiety and restlessness prior to falling asleep,^27^ inspiring us to compare the activity of the mice in the last hour of the active phase. A striking difference between the two groups was evident; the young APP/PS1 mice needed a significantly longer period to fall asleep compared to their littermate controls (Figure 1B, WT 0.098 ± 0.044 a.u., APP/PS1 0.29 ± 0.14 a.u.). Motility-based sleep-wake analysis was extended to include 5- to 6-month-old mice (Figure 1C, WT 0.089 ± 0.039 a.u., APP/PS1 0.24 ± 0.12 a.u.). The 5- to 6-month-old WT mice exhibited a reduction in total activity during the inactive phase compared with 3–4 months (Figures 1B and 1C; Figure S1A; 3–4 months 0.019 ± 0.089 a.u., 5–6 months 0.11 ± 0.04 a.u.), whereas the APP/PS1 mice maintained their activity level during the inactive phase across both age groups (Figures 1B and 1C; Figure S1B; 3–4 months 0.26 ± 0.081 a.u., 5–6 months 0.24 ± 0.10 a.u.). ELISA quantitation at 3 months showed a low baseline level of soluble brain Aβ and no evidence of neuronal degeneration (Figures S1C–S1G). Together, these findings demonstrate alterations in the normal sleep-wake rhythms and confirm the absence of neuronal degeneration in young APP/PS1 mice.

To assess the sleep architecture in detail, electroencephalography/electromyography (EEG/EMG) data were next collected in 3- to 4-month-old mice (Figures 1D–1F and S2). Young APP/PS1 had more waking hours and spent less time in NREM sleep compared to the WT group (Figures 1G and 1H, awake WT 56.3% ± 6%, awake APP/PS1 66.5% ± 4%, NREM WT 37.3% ± 5%, NREM APP/PS1 27.9% ± 3%; Figure S3). These observations were consistent with the delay in falling asleep for the APP/PS1 group noted in the activity tracking analysis (Figures 1B and 1C). Even though no genotypic differences in total duration or latency of REM sleep were noted, the number of REM sleep bouts was significantly reduced in young APP/PS1 mice (Figure 1I, WT 112 ± 7, APP/PS1 87 ± 17; Figure S3). Spectral analysis of NREM and REM sleep showed a trend toward decreased NREM sleep delta power, but only during the active phase, while REM theta power remained unchanged in the APP/PS1 group (Figures S3C and S3D). Spatial memory performance was also comparable between the genotypes (Figure S4). Collectively, young APP/PS1 mice exhibited minor alterations in EEG sleep architecture compared to age-matched littermates and no changes in cognitive performance.

### SWA is increased in the first hour after sleep deprivation

**Figure 2.**
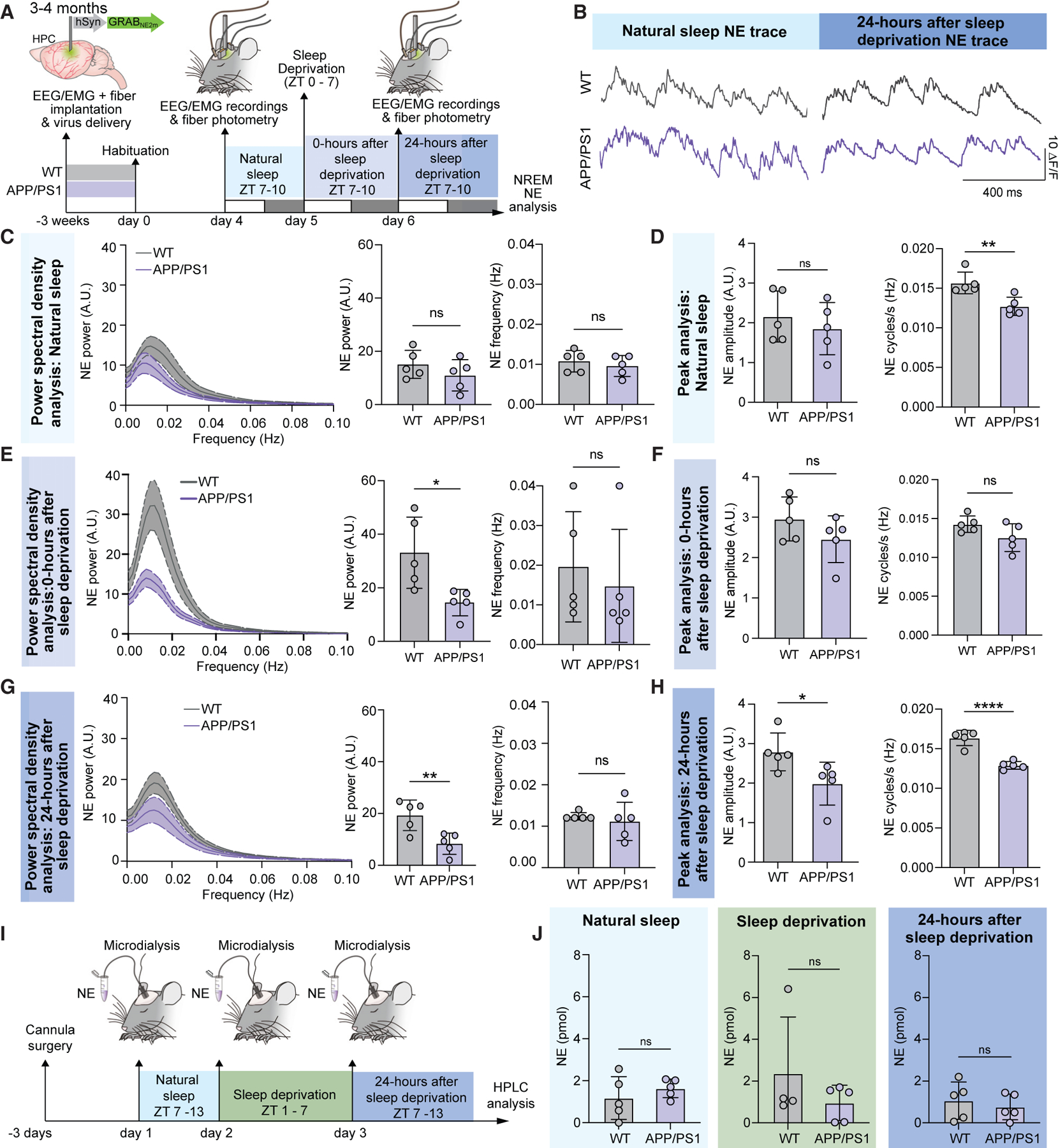
Suppression of NE oscillations after sleep deprivation in young APP/PS1 mice (A) Scheme for fiber photometry experiment with NE biosensor in the hippocampus. (B) Representative trace of fiber photometry NE fluorescent signal during natural sleep and 24 h after sleep deprivation. (C) Power spectral density (PSD) analysis during natural NREM sleep, ns = 0.277, ns = 0.494. Unpaired t test, *n* = 5. (D) NE peak oscillation analysis during natural NREM sleep (3-h interval), ns = 0.482, **p = 0.006. Unpaired t test, *n* = 5. (E) PSD analysis at 0 h interval after sleep deprivation in NREM sleep (3-h interval), *p = 0.02, ns = 0.60. Unpaired t test, *n* = 5. (F) NE peak oscillation analysis at 0 h after sleep deprivation in NREM sleep (3-h interval), ns = 0.19, ns = 0.09. Unpaired t test, *n* = 5. (G) PSD analysis 24-h after sleep deprivation (3-h interval), NREM, **p = 0.009, ns = 0.583. Unpaired t test, *n* = 5. (H) NE peak oscillation analysis 24 h after sleep deprivation in NREM (3-h interval), *p = 0.0383, ****p < 0.0001. Unpaired t test, *n* = 5. (I) Schematic of low-flow microdialysis experiment. (J) Total extracellular NE levels during different brain states, all ns, unpaired t test, *n* = 4–5. PSD traces are presented as the mean ± SEM. Bar graphs show mean ± SD. A.U., arbitrary units.

Prolonged wakefulness leads to an increase in SWA during rebound sleep, which is gradually restored during subsequent recovery sleep.^17^ To validate the reliability of our sleep deprivation model, young APP/PS1 and WT littermates were subjected to sleep deprivation for 7 h (zeitgeber time [ZT] 0–7) (Figure S2). The SWA was assessed from EEG recordings at both 0 and 24 h after sleep deprivation time points (ZT 7–8, both days) (Figure 2A). Sleep deprivation increased the percentage of SWA during the first hour after sleep loss, while the 24-h time point remained comparable to baseline sleep (Figures S5A and S5B, two-way ANOVA; WT sleep to 0 h after sleep deprivation, F(3,24) = 5.0; APP/PS1 sleep to 0 h after sleep deprivation, F(3,33) = 7.7). Interestingly, compared to natural sleep, there was a marked increase in duration of NREM and REM sleep 24 h after sleep deprivation for both genotypes (Figures S5C–S5E, two-way ANOVA; awake, F(1,14) = 74.4; NREM, F(1,14) = 71.6; REM, F(1,14) = 36.7). Together, these observations confirm the efficacy of our sleep deprivation model.

### The NE oscillatory power is comparable in young APP/PS1 and WT mice during natural sleep

Disrupted noradrenergic signaling contributes to AD progression and cognitive decline.^28^ NE is a key regulator of glymphatic flow; the high level of NE suppresses glymphatic activity during wakefulness, whereas the drop in NE levels during sleep facilitates brain clearance.^7^ NE oscillations in NREM sleep are coupled with enhanced memory performance and vascular dynamics,^20,21^ and NE is a potent vasoconstrictor, pointing toward the idea that NE oscillations may drive glymphatic flow during NREM sleep.^23^ We therefore asked whether NE oscillations in APP/PS1 mice differ from those in WT mice. An NE biosensor was expressed under the control of the neuronal promoter hSyn in mouse hippocampus using viral delivery^29^ (Figure S6). This approach enables continuous recordings of NE dynamics. Mice were habituated in the cages 48 h prior to experiments, and recordings were performed during the inactive phase (ZT 7–10) (Figures 2A and 2B; Figure S2). Power analysis of infraslow NE oscillations during continuous recordings over the 3-h window revealed no significant differences between NE power and peak frequency between young APP/PS1 and WT mice (Figure 2C, power, WT 15 ± 5 a.u., APP/PS1 11 ± 6 a.u.; frequency, WT 0.011 ± 0.003 Hz, APP/PS1 0.009 ± 0.003 Hz; Figure S7A). However, NE peak detection during NREM sleep showed a reduction in the number of NE cycles per NREM sleep episode in the APP/PS1 mice (Figure 2D, WT 0.016 ± 0.001 Hz, APP/PS1 0.013 ± 0.001 Hz). Thus, the frequency of NE oscillations in NREM sleep is reduced in young APP/PS1 mice during baseline conditions.

### The NE oscillatory power is reduced in young APP/PS1 mice 24 h after sleep deprivation

Prolonged wakefulness acutely increases Aβ brain burden in WT mice,^2,16^ whereas chronic sleep deprivation increases the deposition of cerebral Aβ plaques in APP/PS1 mice.^2^ Thus, we next asked whether sleep deprivation affects NE oscillation dynamics differently at 0 and 24 h after sleep deprivation in the two groups (Figures 2A and S2). The 0-h after sleep deprivation time point in APP/PS1 animals was characterized by a decreased power of NE oscillations compared to WT controls. The frequency of the NE oscillations did not differ significantly between genotypes, indicating that sleep deprivation primarily suppressed the amplitude of the NE oscillations (Figures 2E, 2F, and S7). Strikingly, a pronounced difference in NE oscillations was noted in APP/PS1 vs. WT mice 24 h after sleep deprivation. Both the amplitude and the frequency of the NE oscillations in APP/PS1 mice were suppressed 24 h after sleep deprivation (Figures 2G and 2H, power, WT 19.3 ± 6 a.u., APP/PS1 8.4 ± 4 a.u.; amplitude, WT 2.8 ± 0.5 a.u., APP/PS1 1.9 ± 0.5 a.u.; frequency, WT 0.016 ± 0.0009 Hz, APP/PS1 0.013 ± 0.0005 Hz; Figure S7). To assess the absolute extracellular NE levels, we additionally performed microdialysis sampling in the hippocampus during natural sleep, sleep deprivation, and a 6-h-long interval 24 h after the sleep deprivation (Figure 2I). The NE levels during natural sleep were comparable between young WT and APP/PS1. Thus, the suppression of NE oscillations was not a result of an increased NE baseline in APP/PS1 mice (Figure 2J, sleep, WT 1.18 ± 1.0 pmol, APP/PS1 1.6 ± 0.4 pmol; sleep deprivation, WT 2.4 ± 2.7 pmol, APP/PS1 0.96 ± 0.84 pmol; 24 h after sleep deprivation, WT 1.1 ± 0.89 pmol, APP/PS1 0.76 ± 0.6 pmol). Taken together, the results show that young APP/PS1 mice exhibited a maladaptive response to sleep deprivation detected as fewer and smaller-scale NE oscillations at 24 h after sleep deprivation. This finding points toward the conclusion that early-stage dysfunction in NE oscillatory dynamics precedes Aβ plaque deposition.

### Sleep loss induces delayed Aβ accumulation in APP/PS1 mice

**Figure 3.**
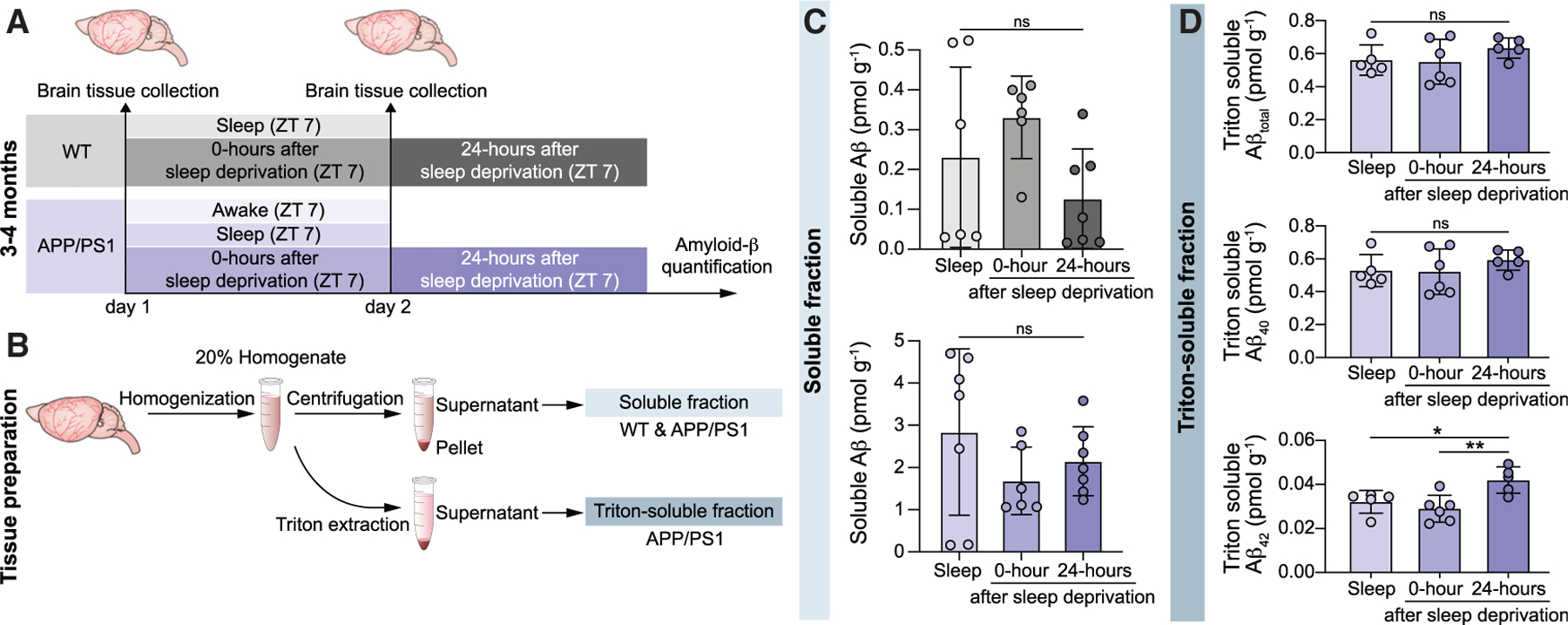
Elevation of Aβ_42_ isoform 24 h after sleep deprivation in APP/PS1 mice (A) Experimental setup for brain tissue collection. (B) Preparation of tissue samples. Brain homogenates were either centrifuged for isolation of the soluble fraction (supernatant) or extracted with Triton X, termed as the Triton-soluble fraction. (C) Concentration of Aβ levels in the soluble fraction measured with ELISA using the 4G8 antibody for WT (top) and APP/PS1 mice (bottom). One-way ANOVA with Tukey’s multiple comparisons test, all ns, *n* = 6–7. (D) Concentration of total Aβ, Aβ_40_, and Aβ_42_ isoforms in the Triton-soluble fraction, quantified with ELISA. One-way ANOVA with Tukey’s multiple comparisons test, *n* = 5–6, *p = 0.046, **p = 0.007. Bar graphs indicate mean ± SD.

Sleep deprivation has previously been linked to an accelerated growth of Aβ plaques in AD brain,^16^ which led us to mapping the effects of sleep loss on the dynamics of Aβ in murine brain using ELISA and western blot (WB).^30,31^ Brain tissue was collected during natural sleep, 0 h after sleep deprivation, and 24 h after sleep deprivation (Figure 3A). Brain homogenates were prepared for evaluation of the Aβ soluble fraction (Aβ monomers) and insoluble fraction (aggregated Aβ) (Figures 3B and S8). As reported before, there was a trend toward increased Aβ monomer concentration in the WT group upon acute sleep deprivation (Figure 3C, one-way ANOVA; WT F(2,16) = 2.7).^2,16^ Surprisingly, in the same setup, APP/PS1 mice exhibited a decreased trend of Aβ monomers, which was not statistically significant (Figure 3C, one-way ANOVA; APP/PS1 F(2,17) = 1.2). Accordingly, a further WB quantification of the insoluble fraction of APP/PS1 brain homogenates was performed to test for evidence of altered Aβ aggregation (Figure S8). As levels of Aβ protein are low in young mice, samples of six animals were pooled for each brain state, and Aβ was immunoprecipitated with the aggregate-specific antibody, mOC31.^30^ For thorough validation of our brain-state paradigm, a natural wakefulness group was added, with matching circadian time points for tissue collection with the sleep group (ZT 7, under reversed light cycle). Remarkably, the WB analysis depicted an elevation of the total brain aggregated Aβ 24 h after sleep deprivation (Figures S8C and S8D). As extracellular plaques in AD patients predominantly contain the Aβ_42_ isoform,^32^ we next tested which Aβ isoforms contributed to the observed aggregations in mice. This analysis confirmed significantly higher concentrations of the Aβ_42_ but not the Aβ_40_ isoform 24 h after sleep deprivation (Figure 3D, one-way ANOVA; total Aβ F(2,13) = 0.99, Aβ_40_ F(2,13) = 0.69, Aβ_42_ F(2,13) = 0.7.2). These findings indicate latent, but sleep loss-induced, Aβ burden along with parallel increase in the Aβ_42_ isoform in the young APP/PS1 mouse brain.

### Glymphatic efflux is impaired in young APP/PS1 mice

Because glymphatic activity is markedly impaired in AD,^33,34^ we next asked whether glymphatic clearance is already suppressed in young APP/PS1 mice prior to Aβ accumulation (Figure S9). Here, a low-molecular-weight water-soluble fluorescent tracer (fluorescein isothiocyanate [FITC]-cadaverine; 640 Da) was injected into the hippocampus of anesthetized mice. This tracer is impermeable to the blood-brain barrier (BBB) and therefore affords broad tissue penetration and distribution via extracellular and transcellular pathways.^35^ Two anesthetic regimens were utilized, one increasing (ketamine and dexmedetomidine; KDex) and the other decreasing glymphatic activity (low-dose isoflurane), based on previous reports.^19,36–39^ Three hours after tracer injection,^40,41^ CSF was collected from the cisterna magna and its content of FITC-cadaverine quantified (Figure S9A). As expected, WT animals had significantly higher content of FITC-cadaverine in CSF under KDex compared to isoflurane anesthesia (Figure S9B, one-way ANOVA; F(3,24) = 9.96, *p* = 0.0002). In contrast, FITC-cadaverine in CSF was low in APP/PS1 mice irrespective of the anesthetic regimen (Figure S9B). This expands upon prior reports of reduced glymphatic clearance in APP/PS1 mice,^34^ revealing that glymphatic efflux is compromised in young APP/PS1 mice before Aβ aggregation.

### APP/PS1 mice retain AQP4 polarization but display reduced microglial activation 24 h after sleep deprivation

Since the AQP4 water channel supports glymphatic flow, its vascular polarization was assessed in the dorsal cortex.^11^ There were no group differences in AQP4 polarization, either during natural sleep or 24 h after sleep deprivation (Figures S10A–S10C, for WT sleep, 87.6 ± 22.1 a.u., and 24 h after sleep deprivation, 77.3 ± 10.8 a.u.; for APP/PS1 sleep, 127.2 ± 23.6 a.u., and 24 h after sleep deprivation, 109.3 ± 9.3 a.u.). Microglial cells actively produce proteolytic enzymes that degrade extracellular Aβ, but also clear Aβ via internalization.^42,43^ Therefore, we investigated whether microglial cells shift toward a more active phenotype during the 24 h after sleep deprivation, which could partially explain perturbed Aβ clearance. Immunohistochemical quantification of percentage of total area of the microglial surface marker Iba1 was compared in natural sleep and 24 h after sleep deprivation in groups of APP/PS1 and WT mice (Figure S10D). This quantification revealed no sleep-state difference in total Iba1 fluorescence for either of the genotypes. Yet, the APP/PS1 mice displayed less total Iba1 fluorescence intensity 24 h after sleep deprivation compared to the WT group (Figure S10D, one-way ANOVA; F(3,20) = 4.4). Morphology analysis in the WT animals showed activation of microglial cells 24 h after sleep deprivation, but not natural sleep, whereas no such phenotypic differences were found in the APP/PS1 group (Figure S10E, one-way ANOVA; density F(3,20) = 3.9, ramifications F(3,20) = 5.8, size F(3,20) = 2.6). These results might reflect a generally less responsive microglial phenotype in APP/PS1 mice.^44,45^ Taking the results together, we find that microglial cells were activated 24 h after sleep deprivation in WT but not in young APP/PS1 mice.

### Extracellular fluid and CSF proteomes show similar composition

**Figure 4.**
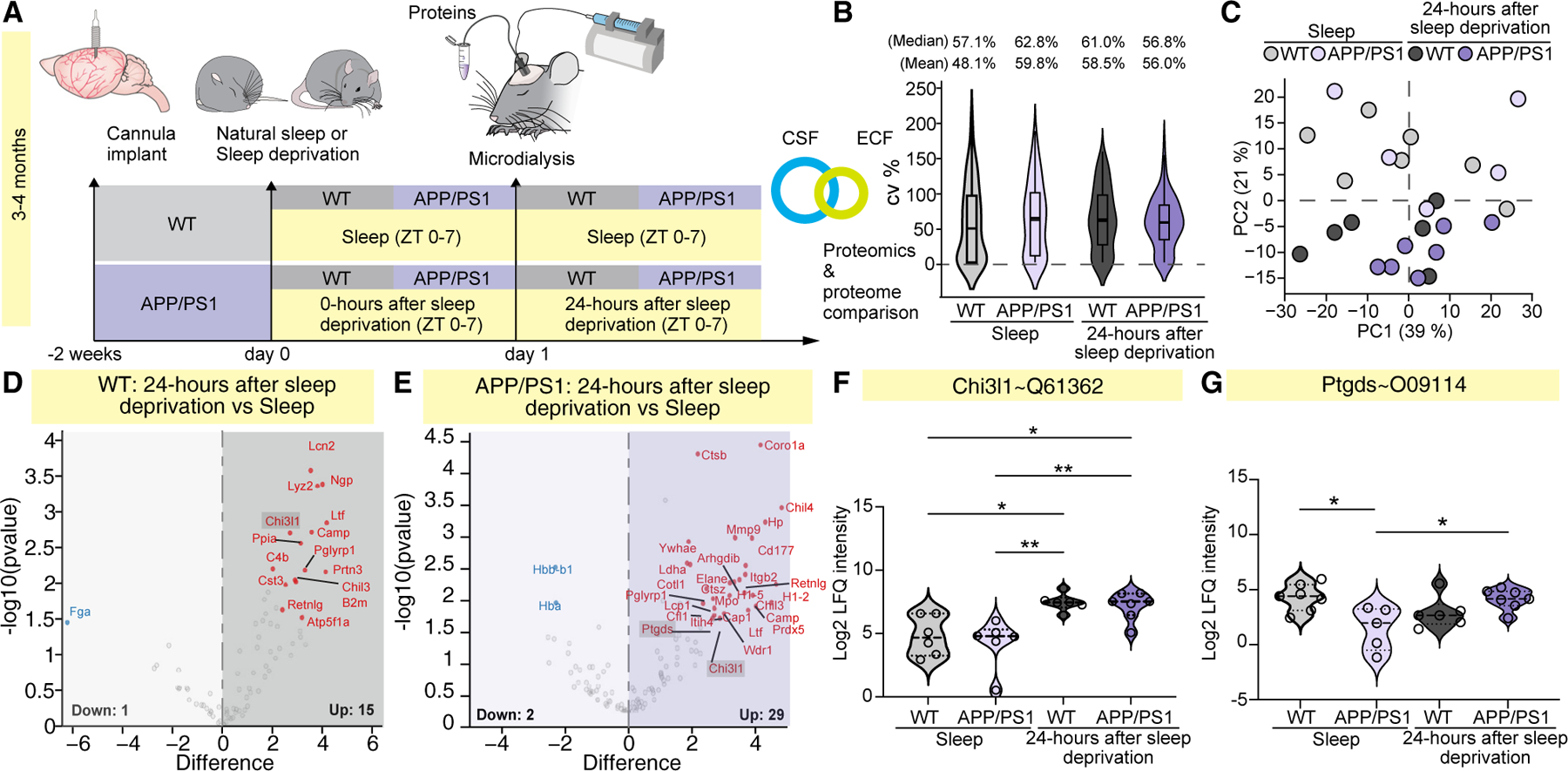
The brain extracellular proteome of WT and APP/PS1 mice is changed 24 h after sleep deprivation (A) Proteomic workflow: microdialysis sampling was performed to characterize proteome overlaps between the extracellular fluid and the CSF. (B) Coefficient of experimental group-wise extracellular fluid sample variation in the original dataset, *n* = 5–7. (C) PCA portraying brain state separation, but no genotypic difference, *n* = 5–7. (D and E) Volcano plots indicating differential protein changes in the (D) WT and (E) APP/PS1 groups between 24 h after sleep deprivation and natural sleep. Differential changes are indicated by red (upregulated) and blue (downregulated) dots with *p* < 0.05 (Student’s t test with permutation correction) and 2-fold change in log2. Gray dots are not significant, *n* = 5–7 mice per genotype. (F and G) Total protein levels of (F) Chi3l1 and (G) Ptgds, expressed as label-free quantification (LFQ) values. One-way ANOVA with Tukey’s multiple comparisons test; Chi3l1 (sleep WT vs. 24 h after sleep deprivation WT, *p = 0.0153; sleep WT vs. 24 h after sleep deprivation APP/PS1, *p = 0.0260; sleep APP/PS1 vs. 24 h after sleep deprivation WT, **p = 0.0033; sleep APP/PS1 vs. 24 h after sleep deprivation APP/PS1, **p = 0.0054) and Ptgds (sleep WT vs. sleep APP/PS1, *p = 0.0109; sleep APP/PS1 vs. 24 h after sleep deprivation APP/PS1, *p = 0.0169), *n* = 5–7 mice. Violin plots show mean ± SD.

**Figure 5.**
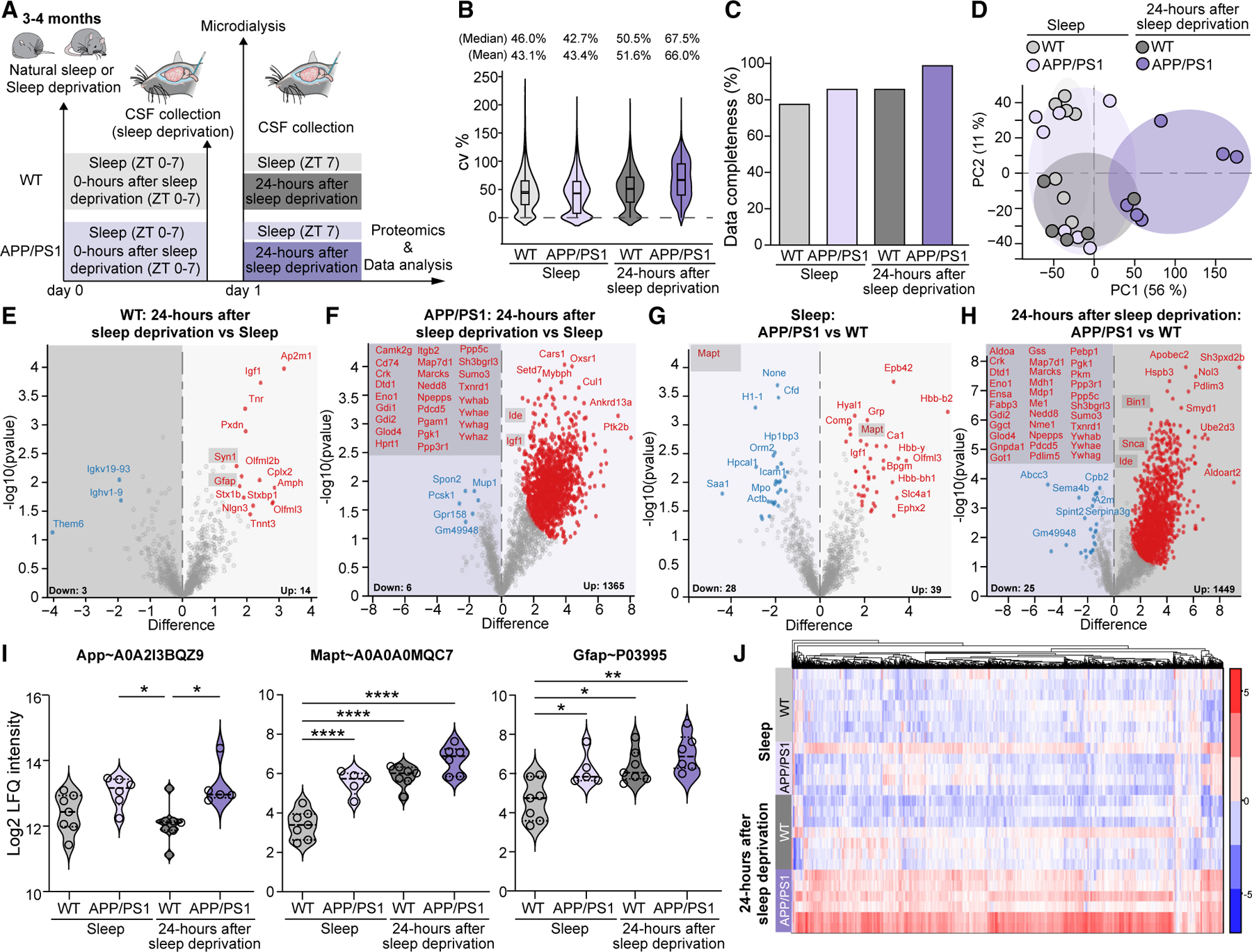
Marked changes in the CSF proteome 24 h after sleep deprivation in young APP/PS1 mice (A) Proteomic workflow for CSF samples. (B) Coefficient of experimental group-wise variation in the original CSF dataset, showing the median/mean and interquartile range, *n* = 5–7. (C) Group-wise comparison of all detected proteins, shown as percentage data completeness, *n* = 5–7. (D) PCA plot reflecting sample stratification across experimental groups. Ellipses around groups are drawn for illustrative purpose only, *n* = 5–7. (E–H) Volcano plots representing protein changes in (E) the WT group, (F) the APP/PS1 group, (G) during natural sleep, and (H) 24 h after sleep deprivation. Differential changes are indicated by red (upregulated) and blue (downregulated) dots, with *p* < 0.05 (Student’s t test with permutation correction) and 2-fold changes in log2. Gray dots are not significant, *n* = 5–7 mice per genotype. Proteins in boxes inside of volcano plot (F and H) overlap with the Higginbotham et al.^62^ proteomic dataset. (I) Total protein levels of App, Mapt, and Gfap proteins between genotypes, expressed as label-free quantification (LFQ) values. One-way ANOVA with Tukey’s multiple comparisons test; App (sleep APP/PS1 vs. 24 h after sleep deprivation WT, *p = 0.0304; 24 h after sleep deprivation WT vs. 24 h after sleep deprivation APP/PS1, *p = 0.0122), Mapt (****p < 0.0001), and Gfap (sleep APP/PS1 vs. sleep WT, *p = 0.0363; sleep WT vs. 24 h after sleep deprivation WT, *p = 0.0103; sleep WT vs. 24 h after sleep deprivation APP/PS1, **p = 0.0022); *n* = 5–7 mice. Violin plots show mean ± SD. (J) Heatmap for LFQ intensities of ANOVA significant proteins, color coded by *Z*-scored values. Blue tones indicate reduced values, whereas red tones are elevated values.

Since we observed changes in NE oscillatory dynamics and accumulation of Aβ 24 h after sleep deprivation in young APP/PS1 mice, we hypothesized that the brain extracellular fluid and CSF proteome are also likely to reflect changes induced by sleep loss. To specifically assess proteins released for export from the brain to the CSF via the glymphatic system, APP/PS1 and WT littermates were implanted with a microdialysis cannula in the hippocampus (Figure 4A). Two weeks later, a microdialysis probe was inserted for sampling of the extracellular fluid during 6 h of natural sleep or at 24 h after sleep deprivation interval. Afterward, the mice were anesthetized for CSF collection (Figure 5A). The extracellular fluid and CSF samples were analyzed using label-free quantification (LFQ) mass spectrometry to study genotypic differences upon sleep loss (Figures 4, 5, and S11). In total, 285 proteins were identified across extracellular fluid samples (Figure S11B). After data filtering and processing,^46^ a set of 134 proteins was selected for further investigation (Table S1). In CSF samples, we detected 4,092 proteins, which is a depth comparable to that of a recent mouse CSF study.^47^ After data filtering and processing,^46^ 2,684 proteins were selected for further examination (Figure S11F; Table S2). To assess the biological properties of the two distinct biofluids, the general compositions of the extracellular fluid and CSF proteomes were compared (Figure S12). Seventy-eight percent of the identified extracellular fluid proteins were also detected in CSF. Since the proteomic depths of the two biofluids differed, we investigated the distribution of molecular weights of the extracellular fluid and CSF proteins to check for any probe cutoff bias in the extracellular fluid and found them comparable (Figure S12B). Based on protein type and cellular location annotations, biofluids had similar compositions (Figures S12C and S12D). Notably, significant proportions of both extracellular fluid and CSF proteomes were derived from the protein pool of the cytosolic compartment (Figure S12D). Overall, these results indicate similar biological profiles between extracellular fluid and CSF.

### Extracellular fluid proteomics enables detection of murine regional brain biomarkers

Given the combinatory microdialysis sampling and unbiased proteomic analysis, the quality of extracellular fluid samples was first evaluated (Figures 4B and S11A). Principal-component analysis (PCA) was indicative of a general difference between natural sleep and 24 h after sleep deprivation, but strong genotype-dependent differences between the samples were absent, potentially due to low proteomic depth (Figure 4C). Samples were not affected by blood contamination, which was evaluated by two common plasma proteins, albumin and hemoglobin alpha^48^ (Figures S11C and S11D). Within the WT group, 16 proteins were differentially regulated between natural sleep and 24 h after sleep deprivation, whereas the APP/PS1 group had 31 protein alterations between the states (Figures 4D and 4E; Table S1). This may indicate that AD mice are more susceptible to sleep loss, which can be detected in the extracellular fluid proteome. Among the regulated proteins, we observed two interesting markers that were affected by sleep deprivation. Specifically, insufficient sleep upregulated cytosolic release of chitinase-3-like protein 1 (Chi3l1 in rodent, corresponding to YKL-40 in human) in both genotypes (Figure 4F). Chi3l1 is a glycoprotein expressed by astrocytes and immune cells and has previously been suggested as a CSF biomarker of physiological aging and AD pathology, although little is known about its specific function.^49–51^ Because genetic deletion of Chi3l1 suppresses formation of amyloid plaques by increasing glial Aβ phagocytosis,^52^ present findings suggest that sleep loss could aggravate Aβ accumulation through the upregulation of this protein. In addition, we found higher prostaglandin-H2 D-isomerase (Ptgds) levels in the APP/PS1 genotype 24 h after sleep deprivation compared to natural sleep (Figure 4G). Given that prostaglandin D2 synthetase determines the production of prostaglandin D2, which is a key factor that promotes natural sleep, our findings of reduced Ptgds levels during natural sleep in the APP/PS1 group may be causally related to the altered sleep pattern observed.^53–55^ Overall, our proteomic analysis of hippocampal microdialysis samples presents an approach for characterizing regional extracellular fluid biomarkers.

### CSF proteome of young APP/PS1 mice is notably altered 24 h after sleep deprivation

Next, the CSF samples were assessed for a representative analysis of the brain exported proteins (Figures 5B and S11E). Most proteins were identified in the APP/PS1 group 24 h after sleep deprivation, while the other groups were comparable, possibly indicating that sleep deprivation may trigger protein release into the CSF of AD mice (Figures 5C and S11F). Again, the number of detected proteins showed no contamination of blood, as validated by two blood markers (albumin and hemoglobin)^48^ (Figures S11G and S11H). Interestingly, however, the hemoglobin but not albumin levels appeared elevated in the APP/PS1 group compared to WT, in both states (Figure S11H). Although the BBB permeability in our pre-depositing APP/PS1 mice appeared intact, we cannot exclude that the protein transport mechanism of specific proteins across the BBB varies between genotypes. Sample stratification was analyzed by PCA, and a clear genotypic difference was observed, where the APP/PS1 group 24 h after sleep deprivation displayed the biggest separation (Figure 5D). Collectively, the quality control and unbiased analysis confirm a sound analytical setup.

**Figure 6.**
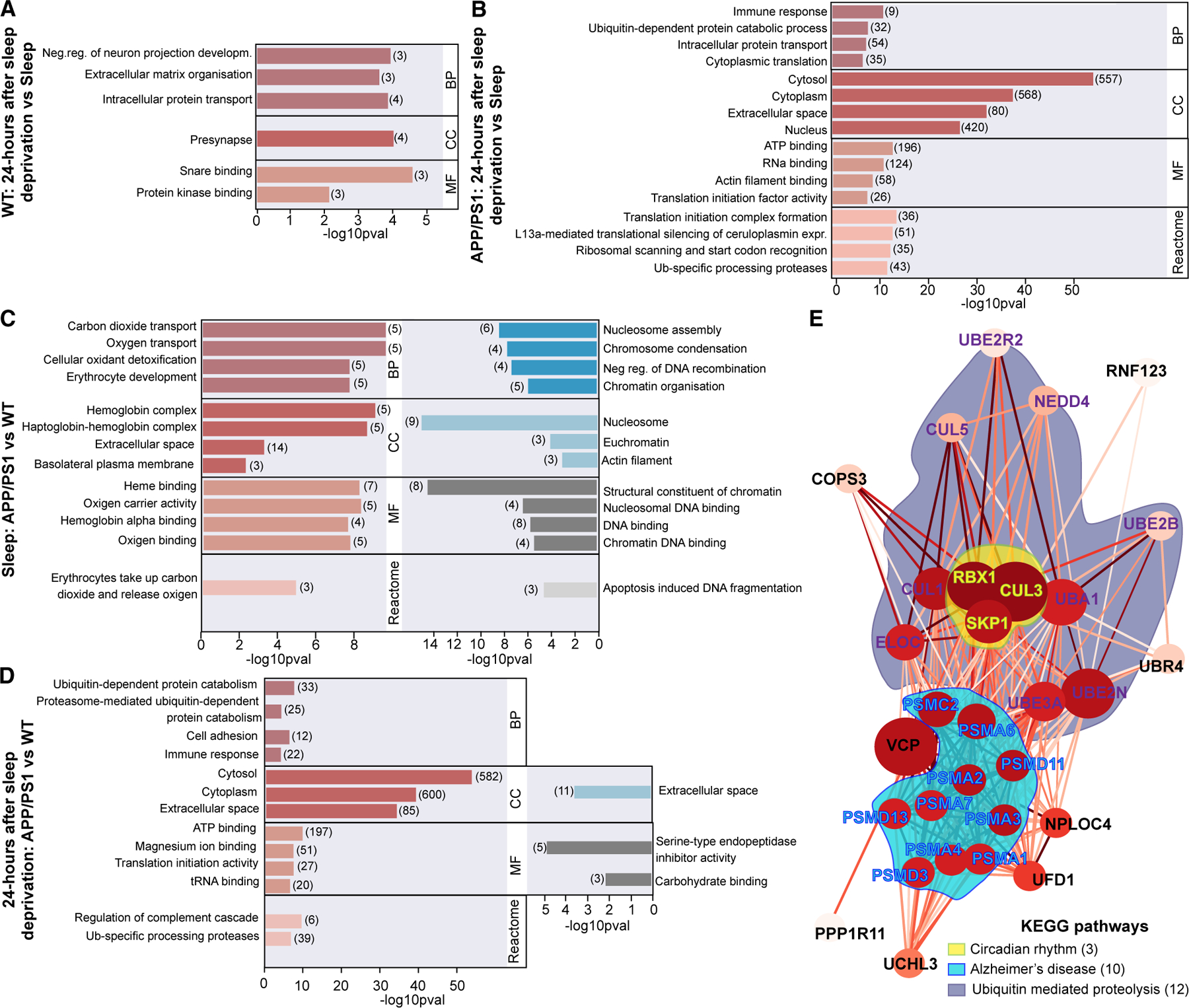
Abnormal ubiquitin proteolysis 24 h after sleep deprivation (A–D) Enrichment analysis of upregulated (red tones) and downregulated (blue tones) protein pathways in (A) WT group between 24 h after sleep deprivation and natural sleep groups, (B) APP/PS1 group 24 h after sleep deprivation and natural sleep, (C) APP/PS1 and WT groups during natural sleep, and (D) APP/PS1 and WT 24 h after sleep deprivation, *n* = 5–7. Numbers of proteins in each pathway are presented in parentheses. (E) Protein interaction network of the top GO terms (33 proteins), based on the STRING database. Dark red tones indicate highly connected nodes and stronger connections between nodes in the network. The KEGG pathways of interest and their members are color coded. BP, biological process; MF, molecular function; CC, cellular compartment.

We examined the differences in protein expression across five experimental groups (Figures 5E–5H and S13; Table S2). Simultaneously, we investigated biological mechanisms linked to these proteome changes across the groups using functional enrichment analysis of the differentially regulated proteins. The top gene ontology (GO) annotations (statements about the function of a particular gene) used were the following: biological processes (BPs), cellular compartments (CCs), molecular functions (MFs), and reactome were explored in detail (Figure 6; Table S2). Between the 24 h after sleep deprivation and natural sleep conditions, 17 proteins were altered in the WT group, whereas 1,371 altered proteins in APP/PS1 mice were identified (Figures 5E and 5F; Table S2). In the WT group, a few enrichment terms of upregulated proteins exhibited associations with ‘‘pre-synapse and extracellular matrix organization’’ and with ‘‘intracellular protein transport.’’ These enriched pathways align with two previous proteomic studies in sleep-deprived healthy rodents (Figure 6A).^56,57^ Furthermore, 7 protein candidates (Amph, Ap2m1, Gfap, Igf1, Stx1b, Stxbp1, and Syn1) out of 17 regulated hits overlapped with brain tissue proteomic findings on sleep-deprived WT C57BL/6J mice,^56^ supporting a solid experimental setup in our study. More specifically, 24 h after sleep deprivation, WT mice (Figure 5E) had upregulated CSF levels of synapsin-1 (Syn1), which is a protein that normally plays a role in maintaining and regulating neurogenesis and synaptic plasticity during sleep.^58^ The elevation of glial fibrillary acidic protein (Gfap), an indicator of astrogliosis, was also noted (Figure 5I).These findings illustrate similarity between the brain tissue and the CSF proteomes and highlight that effects of sleep loss in WT mice are long lasting and remain evident 24 h after sleep deprivation. In the APP/PS1 group, the GO analysis on the 1,371 regulated proteins (Figure 5F) revealed altered pathways that are closely related to ‘‘immune responses,’’ ‘‘ubiquitin-dependent protein catabolism,’’ and ‘‘translation’’ (Figure 6B). These are key processes involved in actively controlling the turnover of proteins and were previously described in AD pathology.^59–61^ Among the significantly regulated proteins, we also identified 26 murine orthologs with increased expression levels, which are listed among the top 65 proteins known to be altered in the CSF of AD patients (Figure 5F; Table S2).^62^ A noteworthy finding was the upregulation of insulin-degrading enzyme (Ide) in APP/PS1 mice subjected to sleep deprivation (Figure 5F). This enzyme is not only involved in insulin homeostasis but also plays a critical role in the degradation of Aβ *in vivo*,^63^ which further supports WB findings on elevated Aβ levels 24 h after sleep deprivation (Figure S8). Similarly, we identified elevated levels of insulin-like growth factor (Igf1) in naturally sleeping APP/PS1 mice compared to WT, which is linked to pathophysiology in AD patients (Figure 5F).^64^ Thus, our CSF findings recapitulated some of the hallmark phenotypic characteristics of AD and sleep deprivation and suggest that these traits are exacerbated upon sleep loss in young APP/PS1 mice.

To further evaluate baseline proteomes during natural sleep, a differential comparison between the genotypes was performed, producing 67 protein candidates (Figure 5G; Table S2). Here, the landscape of enriched GO terms depicted several dysregulated pathways in the APP/PS1 mice compared to WT, mostly related to ‘‘oxygen transport’’ (hemoglobins), but also CCs corresponding to ‘‘nucleosomes’’ (Figure 6C). In agreement, altered nuclear dynamics and modifications of chromatin have been observed in multiple neurological disorders along with AD.^65–67^ Interestingly, we identified one protein candidate previously listed among AD biomarkers, namely microtubule-associated protein tau (Mapt), which was elevated in naturally sleeping APP/PS1 mice compared to WT (Figure 5I).^62^ Together, the overall proteome during natural sleep reflects subtle changes in pathological AD biomarkers already during early stages of amyloidosis.

Potential genotypic differences of protein export into the CSF were also explored immediately after 7 h of sleep deprivation, revealing 73 differentially regulated proteins. Intriguingly, this analysis revealed a downregulation in release of enzymes involved in the metabolism of glutathione, an essential brain anti-oxidant that reportedly has decreased levels in hippocampus and frontal cortex of prodromal AD patients (Figure S13; Table S2).^68,69^ There was a marked reduction of six proteins (Gsta4, Gstm2, Gstp1, Gstm1, Glo1, and Gstm5) in sleep-deprived APP/PS1 mice compared to WT, suggesting a link between insufficient sleep and oxidative stress in the AD brain immediately after sleep loss.

Next, a differential comparison between the genotypes was performed 24 h after sleep deprivation to depict latent modifications in the CSF proteome (Figure 5H). This analysis uncovered no less than 1,474 changed proteins in the CSF of APP/PS1 mice compared to WT. Interestingly, we found widespread alterations in several relevant biological pathways that directly illustrate early consequences of sleep loss in healthy vs. pre-depositing AD brain. Changed BPs due to sleep loss in APP/PS1 animals were associated with ‘‘ubiquitin-dependent protein catabolism’’ (e.g., Psmd13, Psmc2, Psma3, Vcp, Ufd1, Uba1, and Ube2b), ‘‘cell adhesion’’ (e.g., Mybph, Itga5, Itgb1, Pgm5, and Vcl), and ‘‘immune response’’ (e.g., Igkv4–50, Il1rn, and Smad3) (Figure 6D; Table S2). The ubiquitin proteolysis pathway, crucial for regulating ubiquitination and proteasome assembly, was previously implicated in amyloid pathogenesis. In support, previous work showed that ubiquitin-immunoreactive structures and dysfunctional proteasomal processing increase during amyloid pathogenesis.^59,70–72^ Furthermore, 24 h after sleep deprivation, we identified 33 murine orthologs that were reported among 65 human CSF biomarkers in AD for APP/PS1 but not WT mice (Figure 5H; Table S2).^62^ Among regulated hits, we identified an increase in Snca protein, which shows elevated CSF concentrations in early stages of AD and was reported to aggregate in a mouse model of Parkinson’s disease upon sleep deprivation.^9,73^ Similarly, there was a marked increase in amphiphysin 2 (Bin1) protein, which is, next to APOE4, one of the top genetic risk factors for AD.^74^Together, these protein expression changes mark the vulnerability of pre-depositing APP/PS1 mice to sleep loss (Figure 5J). Collectively, the genotype-specific alterations show that CSF proteome changes occur parallel to Aβ aggregation upon 24 h acute sleep deprivation.

### Ubiquitination pathway in APP/PS1 mice is altered 24 h after sleep deprivation

Acute sleep loss leads to elevated levels of ubiquitin protein and modifications in gene expression related to ubiquitin-like protein ligase activity within the cortex.^75,76^ Thus, the most notable enriched term in APP/PS1 mice 24 h after sleep deprivation associated with the GO term ‘‘ubiquitin-dependent protein catabolic process’’ was explored in further detail (Table S2). Overall, this protein group containing 33 members was particularly altered in APP/PS1 mice with insufficient sleep that also exhibited significant elevation of insoluble brain Aβ (Figure 6E; Table S2). Given that Aβ oligomers can impede proteasome function and disrupt neuronal processes,^59,71^ we assessed whether any protein clusters may be linked to AD. Protein-protein interactions revealed a prominent intercluster associated with AD pathology (Figure 6E). Within the protein network, a small group of proteins working downstream of Per1 (Period 1), which contribute to the generation of circadian rhythms, was detected (Figure 6E). Interestingly, previous work reported the general disruption of circadian proteins in hippocampus of middle-aged WT mice.^77^ Thus, young APP/PS1 mice likely manifest sleep- and circadian-disrupted phenotypes, which together increase their vulnerability to AD pathology. Assuming that released proteins reflect their cytosolic content, these altered protein pathways highlight a vital contribution of sleep for the maintenance of intracellular proteostasis. In conclusion, our proteomic data indicate that insufficient sleep triggers aberrant protein metabolism in the APP/PS1 group and link the involvement of the ubiquitin-dependent pathway in this process.

## DISCUSSION

The present study demonstrates that a single episode of sleep deprivation results in delayed parenchymal accumulation of insoluble Aβ in young, pre-depositing APP/PS1 mice. Given that NE is a key regulator of glymphatic activity,^7^ we investigated whether NE oscillation dynamics are altered during NREM sleep in young APP/PS1 mice, both during natural sleep and 24 h after sleep deprivation. Using fiber photometry of an NE biosensor (GRABNE2m) in freely behaving mice, we observed a reduction in NE peaks 24 h after sleep deprivation compared to natural sleep. This was accompanied by a significant suppression of NE oscillation amplitude, not only within 0–3 h interval of sleep deprivation but also persisting 24 h later. These findings suggest that restorative sleep is less effective in APP/PS1 mice. In addition, proteomic analysis of CSF revealed dysregulation of intracellular protein processing 24 h after sleep deprivation, potentially contributing to Aβ accumulation. Proteomic analysis of extracellular fluid identified two regional protein markers, Chi3l1 and Ptgds, that were elevated following sleep loss. In addition, glymphatic efflux of a low-molecular-weight tracer (640 Da) was impaired in young APP/PS1 mice, potentially explaining the delayed accumulation of Aβ. These findings suggest that the inability of APP/PS1 mice to enhance NE oscillations 24 h after sleep deprivation may contribute to glymphatic dysfunction and subsequent Aβ buildup.

Extensive research suggests a bidirectional link between sleep disturbances and AD.^2–4^ Alterations in sleep architecture can precede cognitive decline by decades and are prominent in more than half of all patients.^4^ Furthermore, similar sleep-wake disruptions were reported in an APP/PS1 mouse model upon development of Aβ plaques.^2^ The analysis presented here provides evidence showing that the sleep-wake cycle is already to a minor extent perturbed in young APP/PS1 mice, along with a shift toward longer wakefulness (Figure 1). Since uninterrupted sleep is a prerequisite for efficient glymphatic clearance, it is not surprising that glymphatic flow is suppressed in AD.^78^ Glymphatic clearance is also suppressed in APP/PS1 mice at the age of 6 months, when amyloid starts to deposit.^34^ This observation has been extended to clinical PET imaging of CSF flow, describing reduced brain tissue clearance in AD patients with mild cognitive impairment.^33^ Nevertheless, it remains unclear if sleep disturbances precede glymphatic dysfunction. So far, the glymphatic clearance was assessed only in an APP/PS1 mouse model older than 6 months with the use of radiolabeled molecules.^34^ Here, we assessed glymphatic clearance of a fluorescent tracer from the hippocampus into the CSF, which revealed differences between the genotypes, thus implicating suppression of glymphatic efflux during early stages of Aβ deposition (Figure S9). Based on this, we concluded that changes in sleep architecture and glymphatic flow manifest during the initial disease stage, although we cannot definitively determine which one precedes the other.

NE regulates the glymphatic flow by promoting its activity during sleep, when NE levels are naturally low.^7^ In the brain, this neuromodulator is produced and released by the locus coeruleus, thereby driving the state of behavioral arousal.^79^ However, along with elevated Aβ deposition, dysfunctional noradrenergic signaling is also a key feature of AD.^25,80^ A recent study demonstrated that NE oscillations not only drive sleep-wake transitions but also influence the microarchitecture of sleep.^20,22^ As slow NE oscillations (~0.02 Hz) that occur during NREM sleep promote memory performance,^20^ we compared NE oscillations during NREM sleep in young WT and APP/PS1 animals. During natural sleep, this analysis revealed a general decrease in NE oscillatory cycles in APP/PS1 mice (Figure 2). Interestingly, the WT group exhibited a marked increase in the power of NE oscillations immediately after sleep deprivation as well as 24 h later. In contrast, the NE dynamics within the APP/PS1 group appeared comparable to baseline levels at both 0 and 24 h after sleep deprivation, suggesting that impaired NE dynamics render recovery sleep less efficient (Figure 2; Figure S7). Overall, this indicates that APP/PS1 mice exhibit an impaired ability to boost the NE oscillatory power following sleep deprivation. Interestingly, we found no alterations in baseline levels of extracellular NE, assessed via microdialysis sampling, in either of the genotypes. This was unsurprising in light of the literature^81^ and considering that low-flow microdialysis collection over 6 h, needed for obtaining sufficient sample volume, has limited time resolution.^82^ Hence, microdialysis captures steady-state baseline NE levels, while the fluorescence-based biosensors excel in capturing rapid dynamics of extracellular neurotransmitters.^83^ Importantly, our data suggest that the NE oscillatory power decreases before widespread NE level dysregulation known to occur in AD,^24,25,81^ thus potentially offering an intervention window to enhance NE oscillations prior to irreversible neuronal damage.

A previous study reported that chronic sleep loss leads to greater amyloid plaque deposition in the APP/PS1 mouse model.^16^ Similarly, one night of sleep deprivation increased Aβ accumulation in the hippocampus of healthy subjects.^15^ An elevation in Aβ levels may result from high neuronal activity^16^ and/or reduced glymphatic clearance during wakefulness.^7^ Because APP/PS1 mice exhibited a paradoxical reduction in NE oscillation 24 h after sleep deprivation, we focused on mapping the fraction of soluble Aβ at the same time point. This analysis unveiled that sleep deprivation differentially altered Aβ solubility in the APP/PS1 and WT mouse brains (Figures 3 and S8). While the WT group had the expected decrease in soluble Aβ brain levels during natural sleep and 24 h after sleep deprivation,^2,16^ the soluble Aβ protein in the APP/PS1 brain was altered 24 h after sleep deprivation, as indicated by lower levels of monomeric Aβ. Simultaneously, an increase in the Aβ_42_ isoform exacerbated Aβ aggregation. These findings indicate that upon sleep deprivation, a healthy brain can compensate and subsequently clear Aβ, whereas sleep loss in APP/PS1 mice triggers latent aggregation, because these mice are not capable of initiating restorative processes that remove Aβ during recovery sleep. The observed latency in protein accumulation agrees with a previous human MRI study, which highlighted that recovery sleep did not immediately rectify the clearance of the contrast agent in patients.^84^ Furthermore, another study in human subjects showed decreased glymphatic tracer flow velocity at 24–48 h after sleep deprivation.^85^ Both studies provide valuable insight into the consequences of sleep loss on brain waste disposal, affirming the notion that Aβ deposition is at least in part due to slowed clearance. As we detected no concomitant changes in cortical AQP4 polarization, it is less likely that astrocytes mediate the suppression of glymphatic flow clearance 24 h after sleep deprivation (Figures S10A–S10C). Present findings point to the concept that impaired NE oscillatory dynamics is involved in maladaptive response to sleep loss of the AD brain.

Microglial cells contribute to the removal of extracellular Aβ protein via phagocytosis.^42–44^ Previous studies reported noradrenergic signaling to be essential for sustaining microglial Aβ phagocytosis and motility, such that NE deficiency exacerbated Aβ deposition.^80,86^ We found that sleep deprivation evoked morphological changes only in WT, while APP/PS1 microglia appeared unresponsive (Figures S10D and S10E). We propose that NE fluctuations during sleep might regulate microglial responses and perhaps fail to activate immune cells in early AD stages, in part due to sleep loss. Although our microglial activation was explored in the context of a single sleep deprivation challenge, a more chronic setup might increase their reactivity. Further studies are needed to elucidate if microglial morphological changes are under the modulation of infraslow NE oscillations and perturbed by sleep loss.

After finding that Aβ aggregates 24 h after sleep deprivation in APP/PS1 mice, we proceeded to explore the effects on the global proteome. Previous microdialysis studies have investigated extracellular proteins linked to neurodegenerative diseases.^2,10,87^ The current study couples microdialysis with proteomic analysis to explore the brain extracellular fluid proteome in detail (Figure 4). However, we note limitations of our approach, namely the limited depth of identified extracellular fluid proteome compared to CSF. This is partially due to low sampling recovery rates of proteins from the brain extracellular fluid during microdialysis^88,89^; thus, the difference in proteomic depth between biofluids should be considered when interpreting results. We compensated for the acute inflammation by waiting 2 weeks after probe implantation to collect the samples, but inflammation is unavoidable.^88,89^ Among the detected proteins, 48 were associated with inflammation. Nonetheless, we present the most comprehensive exploratory characterization of extracellular fluid proteome to date and have uncovered interesting proteins, including Chi3l1 and Ptgds, that hold promise for future research in sleep disorders, since their levels were specifically altered after sleep loss.

In CSF, consistent with our hypothesis, the APP/PS1 mice had modified proteome, with particularly increased levels of amyloid-precursor protein as detected by mass spectrometry (Figure 5). Multiple known as well as previously uncharacterized protein biomarker candidates were identified, where 80% were quantified with at least three unique peptides. We found a panel of proteins unique to sleep deprivation (Gsta4, Gstm2, Gstp1, Gstm1, Glo1, and Gstm5), validated several AD markers (e.g., App, Mapt, Bin1, and Igf1), and found numerous proteins reflecting an AD potentiated phenotype^72^ after sleep deprivation (e.g., Chit1, Got1, Ywhag, and Ywhae). Notably, sleep loss altered the release of cytosolic proteins 24 h after sleep deprivation, which was reflected in the upregulation of the ubiquitin-dependent catabolism pathway in the CSF proteome (Figure 6). This pathway is vital for sustaining cellular proteostasis and regulating the breakdown of damaged or excessive proteins via the proteasome but becomes dysfunctional in aging and neurodegenerative disorders.^59,70,90^ Both *in vitro* and animal work shows evidence that high levels of Aβ inhibit ubiquitin-dependent protein degradation and might impair proteasome activity in the context of aging.^71,91,92^ The exact ratio of proteinaceous waste clearance via the ubiquitin-proteasome or glymphatic system remains unknown, although the two pathways control the intercellular and the extracellular protein pool, respectively.^7,93^ In addition, intracellular bulk degradation of larger aggregated proteins and damaged cellular organelles, which are too large for proteasome processing, occurs through the autophagy-lysosomal pathway.^61^ It is hard to make predictions regarding the ratio between intra- and extracellular protein removal, as these proteins likely differ across brain regions and are dependent on age and state of brain activity. Nevertheless, our proteomic findings suggest that acute sleep deprivation prompts the overall need for protein removal in young APP/PS1 mice, as indicated by the upregulation of released core building units of the proteasome (Psma1, Psma4, and Psma7) and ATPase subunits (Psmc2, Psmd3, and Psmd13), which are mechanically involved in protein degradation. One hypothesis is that an increased intracellular protein clearing mechanism in these mice acts to compensate for early dysfunction of glymphatic clearance. While our findings demonstrate that changes in global brain proteostasis occur alongside Aβ aggregation, after a single sleep deprivation, chronic sleep disturbances may amplify the vulnerability to brain proteinopathies. Thus, we propose that future strategies targeting the enhancement of NE oscillation dynamics could offer a therapeutic approach for early-stage AD, aiming at improving glymphatic activity.

### Limitations of the study

While our approach successfully demonstrates the effects of sleep loss on the suppression of infraslow NE oscillations, leading to proteostasis in the APP/PS1 murine model, further studies are needed to directly assess how sleep deprivation impairs glymphatic flow. Therefore, performing *in vivo* single-photon emission computed tomography (SPECT) imaging of CSF flow will be crucial for a more comprehensive understanding of sleep disturbances and early amyloid pathology.

Furthermore, it will be important to determine whether the changes observed in proteomes of extracellular fluid and CSF merely reflect intracellular changes and can serve as clinical biomarkers or if these altered biofluids actively contribute to the disease process and offer potential therapeutic targets. For example, would the infusion of sleep-deprived CSF from APP/PS1 mice into rested WT mice have detrimental consequences and, in turn, could these then be prevented by depletion of key proteins or metabolites? Additional studies should aim to determine whether ubiquitin-related markers within CSF have functional effects and if tailored drugs could rescue proteasomal dysfunction during neurodegeneration.

## RESOURCE AVAILABILITY

### Lead contact

Requests for further information and resources should be directed to and will be fulfilled by the lead contact, Maiken Nedergaard (nedergaard@urmc.rochester.edu).

### Materials availability

This study did not generate new unique reagents.

### Data and code availability

- All data reported in this paper will be shared by the lead contact upon request.
- MATLAB code for NE oscillation analysis is available via GitHub: https://github.com/MieAndersen/Sleep_NE_APP_PS1/tree/main.
- The mass spectrometry proteomics data have been deposited with the ProteomeXchange Consortium via the PRIDE partner repository with the dataset identifier PXD054763.
- Python code for proteomic analysis is available via GitHub: https://github.com/MannLabs/CKG
- Any additional information required to reanalyze the data reported in this paper is available from the lead contact upon request.

## STAR★METHODS

### EXPERIMENTAL MODEL AND STUDY PARTICIPANT DETAIL

#### Mice

Unless stated otherwise, 3 to 4-month-old heterozygous transgenic (APP/PS1) mice and their wild type (WT) controls of both genders were used in all experiments. The APP/PS1 mice contain human transgenes for APP (Swedish mutation) and PSEN1, both under the Thy1 promoter. Specifically, the mouse line B6;C3-Tg(APPswe,PSEN1dE9)85Dbo/Mmjax on a C57BL/6;C3H genetic background does not exhibit seizure activity (#034829-JAX). Its mutations are associated with early onset AD^26^. Within the study, we used young animals, before the start of plaque deposition. Mice were kept under 12h inactive/ 12h active phases, with lights on/off at 7:00 am/7:00 pm. All experiments were approved by the Danish Animal Inspectorate and carried out at the Center for Translational Neuromedicine, University of Copenhagen in accordance with the European directive 2010/63/EU. The current study followed the ARRIVE guidelines.

### METHOD DETAILS

#### Ethical declaration

All the animal experiments were approved by the local research ethics committee (Department of Experimental Medicine, University of Copenhagen) and conducted in accordance with the Danish Animal Experiments Inspectorate. Experimental design ensured minimal animal numbers.

#### Tracking animal activity

For the cross-evaluation of manual sleep scoring, the natural home cage behavior of the animals was recorded and tracked. During the experiment, mice were housed individually, with habituation to the new home cages for a minimum of 12-hours. Subsequently, the natural home cage activity was recorded for 48-hours with an infrared camera. Following this procedure, animals returned to their original group housing. EthoVision XT 11.5 (Noldus) served to track activity in the acquired videos.

#### Histology for mouse strain evaluation

Before staining, immersion fixed, and frozen right hemispheres were cut into serial 25 μm-thick coronal sections (Vibratome VT1200 Leica, Biosystems). The sections were collected into 12-well plates containing cryoprotectant solution (35% ethylene glycol, 25% glycerol in 1x PBS) and stored at −20 °C until further use. For immunohistochemical staining of human Aβ protein, we used the polyclonal antibody 6E10, as described previously ^30^. We took every 12th section for the counting of amyloid plaques.

#### EEG surgery

During surgery, anesthesia was maintained at 1–2 % isoflurane and mice were fixed onto a stereotaxic frame (Elevated U-Frame Stereotaxic Instrument, Harvard Apparatus). Subcutaneous preoperative buprenorphine (0.05 mg/kg) and lidocaine (0.03 mg/kg) was administered at the scalp incision site. Three burr holes were drilled in the skull using an electric drill (Tech2000, RAM Microtorque 45,000 rpm), according to stereotactic coordinates relative to bregma. Mono fiberoptic cannulae (400 μm, 0.48 NA, Doric Lenses) attached to a 2.5mm diameter metal ferrule were pre-coated with a mixture of silk fibroin and AAV9hSynGRABNE2m (virus provided by Yulong Li) ^29^, and were then implanted in the hippocampus at the following coordinates: M/L: −3.20 mm, A/P: −3.30 mm, D/V: −4.00 mm. Two 0.8 mm low impedance stainless steel screws (NeuroTek) were then implanted in the burr holes above the frontal cortex and cerebellum, serving for electroencephalography (EEG). An electromyogram (EMG) electrode made of silver wire (W3 Wires International) was inserted into the trapezius muscle. After the surgery, we administered carprofen (5 mg/kg) subcutaneously, with continued analgesic treatment for two days post-operatively. The animals had a minimum of two weeks for surgerecovery prior to experiments.

#### EEG recordings

For the sleep cycle evaluation, we acquired sleep recordings in 48-hour periods, preceded by at least 48-hours of habituation to the recording chamber (ViewPoint Behavior Technology). The sleep recordings combined with fiber photometry were acquired over a 3-hour period during the inactive phase (ZT 7–10). The EEG and EMG signals were amplified (National Instruments, 16-channel AC amplifier, model 3500) and filtered (EEG signal: high-pass at 1 Hz and low-pass at 100 Hz; EMG signal: high-pass at 10 Hz and low-pass at 100 Hz), and power line noise was reduced using a notch filter of 50 Hz. Signals were digitized using a multifunction I/O DAQ device (National Instruments, USB-6343) and sampled at a rate of 512 Hz. We obtained simultaneous video recordings (FLIR Systems). Acquisition of sleep recordings and sleep scorings were performed with SleepScore software (ViewPoint Behavior Technology). Automated sleep deprivation (SD) was performed using shaker software that is compatible with the recording chambers (ViewPoint Behavior Technology), following a protocol employing a pattern of randomized pulses (high intensity duration: 10–30 ms, total duration: 600–1000 ms, delay: 100–200 ms). For the sleep cycle evaluation, we manually created hypnograms from the EEG traces in 5-second epochs, and for the sleep recordings combined with fiber photometry in 1-second epochs. Actimetry and video were used to help score vigilance states, as follows: ‘‘wake’’ defined by high muscle tonus and a high frequency, low amplitude EEG pattern, ‘‘NREM sleep’’ showing no muscle tonus and a low frequency, high amplitude EEG, and ‘‘REM sleep’’ showing no muscle tonus and high frequency, low amplitude EEG. For the 48-hour long sleep recordings, we calculated the average power of specific frequencies from the power spectrum frequencies in each state (wake, NREM, and REM). Delta and theta power for either NREM or REM were calculated as the mean value of the fast Fourier transform (FFT) power in frequency bands 1–4 Hz and 4–8 Hz, respectively. For the 3-hour sleep recordings combined with fiber photometry, both for baseline and 0-hours or 24-hours after sleep deprivation, we calculated average power of specific frequencies of NREM during the first hour of the recording by applying Welch’s method on the EEG traces from NREM sleep (Matlab pwelch function).

#### Immunohistochemical validation of fiber photometry viral expression

Validation of optic implant location and viral expression was performed via immunostaining of the brain sections. Animals were placed under deep anesthesia using ketamine (10 mg/ml) and xylazine (1 mg/ml) mixture for transcardial perfusion with 1x PBS followed by 4% formalin buffer. Brains were harvested and post-fixed in 4% formalin buffer overnight and transferred to 1x PBS until sectioning (stored at 4 °). Next, we prepared 60 μm-thick coronal sections surrounding the implant locations using a vibratome. Sections were then blocked in PBS with 5% goat serum and 0.1% Triton X-100 at room temperature for 2 hours, before overnight incubation with custom-made primary anti-GFP rabbit antibody (1:500) at 4 °C ^94^. After washing, sections were incubated with anti-rabbit Alexa Fluor 488 (ThermoFisher Scientific, #A-11034) at room temperature for 2 hours and then mounted on glass slides using DAPI-containing mounting medium (Vectashield, H-1200). Images of whole brain slices (5×5 tiles) were acquired using a Nikon Ni-E Eclipse microscope equipped with a DS-Fi3 camera at 4x magnification and stitched together in the NIS-Elements imaging software (Nikon).

#### Glymphatic efflux study

Under light anesthesia (0.8–1.2% isoflurane), mice aged 3–5 months were implanted with a guide cannula (mouse guide cannula 4.5 mm, Bilaney) into the hippocampus region (coordinates: M/L: +2.7, A/P: −3.0, D/V: −2.3). After 2–3 days of recovery from surgery, animals were used for efflux experiments with parenchymal tracer infusion. Here, mice were anesthetised with ketamine (100 mg/kg) and dexmedetomidine (0.5 mg/kg) (KDex) i.p., or with low concentration isoflurane (0.5–0.8%). Following infusion with 2 μL cadaverine tracer (Alexa Fluor 488 cadaverine, 1%) at the rate of 0.2 μL/min for 10 min. The tracer was allowed to circulate in the brain for 3-hours. Then, we collected CSF samples as described above, with 1:300 dilution in aCSF for quantitation of fluorescence signal with a microplate reader (SpectraMax iD5, MolecularDevices).

#### Fiber photometry

Fiber photometry combined with EEG/EMG recordings was performed during the inactive phase of natural sleep (ZT 7–10) or 0-hours and 24-hours after sleep deprivation (ZT 7–10), following 4-days of habituation to the cages. We used one set of excitation LEDs (465 nm and 405 nm, Tucker-Davis Technologies), which was connected to a minicube (Doric Lenses) using an attenuator patch cord (400-μm core, NA 0.48, Doric Lenses). The minicube optics allows the monitoring of fluorophores using dichroic mirrors and cleanup filters, which we selected to match the excitation and emission spectra. The RZ10-X real-time processor (Tucker-Davis Technologies) served to drive the LEDs. The 465-and 405-nm excitation lights were delivered through the same patch cord to the hippocampus; The 465 nm wavelength served to excite GRABNE2m fluorescence, while the 405 nm excitation served as an excitation isosbestic wavelength, which helped to correct for bleaching and signal fluctuations due to movement. The 465-nm/405-nm excitations were sinusoidally modulated at 531 Hz/211 Hz. Fiberoptic patch cords (400-μm core, NA 0.48, Doric Lenses) established a light path between the minicubes and the animals. Zirconia sleeves were employed to attach the fiberoptic patch cords to the fiber implants in the animals.

For signal recovery, each of the two modulated signals generated by the LEDs was independently retrieved using standard synchronous demodulation techniques, which were implemented on the RZ10-X real-time processor at a sampling rate of 1,000 Hz. The Synapse software (Tucker-Davis Technologies) was utilized to control the signal processor and synchronize fluorescent signals with video and EEG/EMG signals through incoming or outgoing TTL pulses. Subsequently, the files were exported to MATLAB R2020a software (MathWorks) for analysis. ∆F/F calculations were conducted based on the fitted 405-nm signal or by using the median of the fluorescence signal itself. To achieve the 405 nm fitting, we first normalized the 465-nm signal and the isosbestic 405 nm channel using a least squares method (MATLAB polyfit function) to determine the slope and intercept required to generate a scaled 405-nm channel:

scaled405nmchannel=a*405nmchannel+b

Next, ΔF/F was calculated by subtracting the fitted control channel from the signal channel:

ΔF/F(%)=(signal465nm-scaled405channel)*100/scaled405channel

To evaluate the oscillatory patterns of NE during NREM sleep, power spectral analysis was applied to the NE signal. In short, NE traces from NREM bouts were detrended and centered using a polynomial fit, and power spectral density (PSD) was determined for each bout using Welch’s method (MATLAB pwelch function). The NE amplitude was calculated as the maximum value of the averaged PSD plots. The oscillation frequency of NE represents NE peaks occurring during NREM episodes in 3-hour long fiber photometry recordings (MATLAB findpeaks function with 10 s minimum interval between peaks and 0.5 ΔF/F (%) minimum amplitude).

#### Manual sleep deprivation

Sleep deprivation for microdialysis experiments was imposed at the beginning of the inactive phase (ZT 0–7), for a total duration of 7 h. The animals were kept awake in their housing cages by gentle handling and exposure to novel objects, aiming to engage them in exploration activity. The 24-hours after sleep deprivation had undergone sleep deprivation on the previous day, while 0-hours sleep deprivation on the same day.

#### Low flow microdialysis and high-performance liquid chromatography

Microdialysis low flow rate (0.1 μL/min) sampling of NE was performed in the hippocampus region (A/P: −3.0 mm, M/L: −3.0 mm and D/V: −3.0 mm) with the 7 kDa cut-off probes (CMA 7 Microdialysis Probe, 2 mm membrane length, Harvard Apparatus). Sampling was performed during natural sleep (ZT 7–13), during sleep deprivation (ZT 1–7), and during the six-hour interval 24-hours after sleep deprivation (ZT 7–13). Microdialysis guide cannulas (CMA 7 microdialysis probe guide, Harvard Apparatus) were implanted 14 days before the sampling experiment. Animals were well-habituated to the sampling cages. During the 7-hours sampling period, we infused artificial CSF (119 mM NaCl, 3.5 mM KCl, 1.0 mM CaCl2, 0.8 mM MgCl2, Hepes 10 mM, pH 7.2, dissolved in dH2O), with discarding of the first 1-hour sample to avoid artifactual NE signal associated with the probe insertion. Samples were snap frozen and the NE measured by HPLC with electrochemical detection. In brief, we used a Prodigy 3-μm ODS-3 C18 (DA 2×100 mm, particle size 3 μm, Phenomenex, YMC Europe) analytical column with mobile phase (55 mM sodium acetate, 1 mM octanesulfonic acid, 0.1 mM Na2 EDTA and 8% acetonitrile, pH3.2) delivered at 0.15 ml/min. Equal sample volumes (10 μl) were injected, with amperometric detection (Antec DECADE) at 0.8V. Final peak areas were assessed by LC solution software (Shimadzu).

#### Brain homogenates and Aβ quantification

Snap-frozen brain tissue hemispheres were weighed and homogenised in 20% (w/v)1x PBS solution. Samples were processed with Precellys Evolution homogeniser in 2 mL tubes (2.8 mm steel balls, Bertin Technologies SAS) in 3 cycles for 30 s with 10 s breaks between and were immediately transferred to ice after homogenization. Next, we centrifuged the samples at 21,000 g for 60 min at 4 °C (soluble fraction) or extracted with 2% Triton X-100 in PBS (Triton-soluble fraction), as previously described ^30^. Both supernatant aliquots were stored at −80 °C until their use for Aβ quantification. Levels of Aβ protein in the supernatants, designated as the soluble or Triton-soluble fractions, were measured with an electrochemiluminescence-linked immunoassay Meso Scale Discovery (MSD) 4G8 panel, as described previously ^31^. Briefly, supernatant fractions were diluted 1:2 with a standard kit buffer (Dilutent 35, MSD) to fit within the recommended linear assay range. A commercially available kit (V-PLEX Aβ Peptide Panel 1, 4G8) was used according to the manufacturer’s protocol. Precoated plates were blocked (1 h with Dilutent 35 buffer) and washed 3x with PBS (containing 0.05 % Tween-20). The samples were incubated with detection antibody (SULFO-TAG-labelled) for 2 h at room temperature. Upon washing and adding MSD read buffer T, the plates were read on the SECTOR Imager 6000. Subsequent data analysis was conducted with the MSD DISCOVERY WORKBENCH software v.2.0. For each plate, reference samples were included as controls. For strain assessment, Aβ proteins in the brain homogenates were assessed as described above. NfL concentrations in CSF were determined using the highly sensitive Simoa^™^ NF-Light Advantage assay Kit (Quanterix, Billerica, MA). Murine CSF samples were pre-diluted 1:100 in sample diluent and measured in duplicate on a Simoa HD-1 Analyzer (Quanterix, Billerica, MA) according to the manufacturer’s instructions.

#### Western Blot with IP

The pellets from 20% brain homogenates were used for western blot quantitation of Aβ levels in the insoluble fraction. First, we pooled the samples from 6 APP/PS1 animals in the same brain state for immunoprecipitation (Dynabeads Protein G immunoprecipitation Kit, Thermo Fischer Scientific) with the anti-vascular Amyloid 1–42 antibody (mOC31, Abcam), according to the manufacturer’s protocol. Resultant samples were separated with SDS-PAGE on 12% Bis-Tris protein gels (Thermo Fischer Scientific) using NuPAGE MES SDS running buffer (Thermo Fischer Scientific). Transfer from the gels onto pre-assembled nitrocellulose membranes (Trans-Blot Turbo Midi 0.2 um, Bio-Rad) was performed with the Trans-Blot Turbo transfer system (Bio-Rad) and NuPAGE transfer buffer (Thermo Fischer Scientific). Next, the membranes were blocked with 5% milk in PBS for 45 minutes and then incubated with the anti-Aβ 4G8 primary antibodies (1:2,000, Thermo Fischer Scientific) overnight at 4 °C. After 3× 10 min washing with PBS and Tween-20, the goat anti-mouse IgG antibodies tagged with the HRP (1:10,000, BioSite) were applied to the membrane for 2 h incubation at room temperature. Membranes were imaged with ChemiDoc Imaging System (Bio Rad) after 3 min incubation with the PierceTM ECL western blotting substrate (Thermo Fischer Scientific). Total protein load was assessed with the anti-GAPDH antibody (Thermo Fischer Scientific). Protein intensities were analysed using ImageJ/Fiji software (https://imagej.nih.gov/ij/download.html).

#### Immunohistochemistry

Frozen and fixed brain hemispheres were cut into 25 μm-thick coronal cryostat sections (Thermo Scientific), which were collected in 12-well plates filled with cryoprotection solution (30% glycerol and 25% ethylene glycol in PBS). The brain sections were stained for microglial Iba1 (rabbit polyclonal anti-Iba1 antibody, 1:1000, Wako Chemicals) and astrocyte AQP4 (mouse anti-AQP4 antibody, 1:1,000, Abcam) marker. In brief, sections were washed 3x with PBS and blocked for 2 h in normal goat serum (5% in PBS and 0.3% Triton-X 100) at room temperature. Primary antibodies were used for overnight staining at 4 °C. After washing with PBS, fluorescent staining was performed for 2 h using secondary antibodies conjugated with Alexa Fluor 488 and 568 (both Alexa Fluor, 1:500, Thermo Fisher Scientific). Afterwards, tissue sections underwent DAPI (4’,6-diamidino-2-phenylindole, Thermo Fisher Scientific, #62248, 1:1,000) staining prior to mounting.

#### Microglial quantification and AQP4 polarization index

For the Iba1 marker, was used confocal microscopy (Nikon Eclipse Ti, Japan) at 20x magnification (Plan Fluor 20X/0.75) with a 20 μm z-stack. For each animal, three confocal z-stack images in the cortex region were taken as biological replicates. Image analysis of microglia was performed with ImageJ2 for MAC (ver. 2.3.0/1.53q, https://imagej.net/), with binarization into black-white stacks. Maximum projection intensities were first thresholded, and images subsequently analyzed with the integrated ‘‘particle analysis tool’’. Particles measuring between 30–90 μm^2^ were counted as microglial cells, and particles between 5–30 μm^2^ as microglial ramifications. Particles measuring below 5 μm^2,^ were not included in the quantification. For each image, we then calculated the total number of cells per area and the number of ramifications per cell. Vascular polarization of the AQP4 marker was assessed in the dorsal cortex due to its high regional glymphatic inflow. Mounted coronal sections were imaged with a confocal microscope (Nikon Eclipse Ti, Japan) at magnification of 60x (Plan Fluor 60x/1.40) with 20 μm z-stacks. In ImageJ software, we analyzed the maximum intensities of the signal to quantify the polarization around blood vessels^95^. In short, we drew line ROIs across multiple blood vessels in the cortical region and assessed their fluorescent signal. For each image, selected at least 12 and at most 30 blood vessels. After subtracting 5 μm of the background signal from the line peak, we determined a polarization index, and express the values relative to the highest value.

#### Microdialysis sampling of brain extracellular fluid and CSF collection

The microdialysis guide cannulas (500 kDa, CMA 7 Microdialysis Probe Guide, Harvard Apparatus) were implanted 14 days before the experiment, to minimize the inflammatory responses. To assess extracellular brain proteome, we undertook microdialysis sampling in the hippocampus region (A/P: +3.0 mm, M/L: −3.0 mm and D/V: −3.0 mm). After habitation of the mice to the sampling chambers, we perfused the microdialysis probes (CMA 7 Microdialysis Probe, 2 mm membrane length, Harvard Apparatus) with artificial CSF (119 mM NaCl, 3.5 mM KCl, 1.0 mM CaCl2, 0.8 mM MgCl2, Hepes 10 mM, pH 7.2, dissolved in dH2O) at a constant flow rate of 1.0 μL/min. Samples collected during the first hour were discarded to avoid potential blood contamination due to probe insertion. After 6 h sampling, mice were anesthetised with ketamine (10 mg/ml) and xylazine (1 mg/ml) i.p. in 0.9% saline. CSF was collected from cisterna magna with a 20 μL pipette tip, after puncturing the dura with a sharp needle, as previously described ^30^. After collection, CSF was centrifuged at 2,000 g for 10 min and aliquoted for further analysis. Blood was collected into EDTA-coated 1.5 mL tubes via intracardiac puncture, immediately centrifuged at 10,000 g for 10 min, and the supernatants stored at −80 °C. Mice were transcranially perfused with 1x PBS and the visceral organs dissected out. After separating the brain hemispheres with a midline sagittal cut, the right hemispheres were fixed in 4% paraformaldehyde in 1x PBS for 48 h at 4 °C, cryopreserved in 30% sucrose, and snap frozen in 2-methylbutane at −40 °C. The left hemisphere was snap frozen on dry ice immediately after dissection and stored on −80 °C until further immunohistochemical analysis.

#### Sample preparation for mass spectrometry

Cerebrospinal fluid (CSF) and extracellular fluid (ECF) samples were processed for analysis by mass spectrometry, with randomization of each set of samples to avoid batch effects. A detergent-free lysis buffer (‘E buffer’; 100 mM Tris, 40 mM CAA, 10 mM TCEP, pH 8.5) was used for lysis for each of the sample sets. CSF and ECF samples were processed almost identically, as follows: 10 μL raw CSF was mixed into 40 μL lysis buffer, whereas 40 μL ECF was mixed into 60 μL lysis buffer, and both sample sets were then heated at 95 °C for 10 minutes on a shaker. Next, 1:100 (w/w) trypsin (Sigma Aldrich, #T6567) and LysC (WAKO, #129–02541) enzyme was added to each sample, which were allowed to digest on a shaker at 37 °C for two hours, whereupon the reaction was quenched by acidification using 1% trifluoroacetic acid (TFA). The samples were then desalted on SDB-RPS Stagetips (Sigma Aldrich), eluted using 1% ammonia in 80% acetonitrile, and finally evaporated at 45 °C under reduced pressure to dryness. After resuspension in A* buffer (5% acetonitrile, 0.1% TFA), 500 ng peptide portions of each sample were loaded onto an Evotip (Evosep Biosystems) according to the manufacturer’s instructions.

#### Mass spectrometry acquisition

ECF and CSF samples were both run on an Evosep One LC system (Evosep Biosystems) connected to a timsTOF Pro mass spectrometer (Bruker) by a 15 cm performance column (Evosep Biosystems; 150 μm inner diameter, 1.5 μmC18 beads) kept at 40 °C using a 30 samples-per-day (‘SPD’) standard gradient method. The mass spectrometer was operated with positive ion polarity and capillary voltage 1750 V, with 100% duty cycle and 100 ms ramp time. Samples were run with a diaPASEF method with 20 isolation windows of varying widths (between 20 and 200 m/z) covering a mass range of 400 to 1200 m/z and ion mobility range 0.80 to 1.30 V*s/cm-2. The collision energy was set to rise with greater ion mobility, from 20 eV at 0.60 V*s/cm-2 to 59 eV at 1.60 V*s/cm-2, and cycle time was estimated at 1.16 s. Each set of DIA sample files was processed in Direct DIA mode in the software package Spectronaut 16 based on a mus musculus proteome from Uniprot (downloaded 06-04-2021). The analysis settings allowed peptides containing from 7 to 52 amino acids, with a maximum of two missed cleavages, N-terminal acetylation, and methionine oxidation as variable modifications, and 1% false discovery rate (FDR).

#### MS bioinformatic analysis and visualizations

Proteomic data processing (ECF and CSF) including normalization, filtering, statistical, and enrichment analysis, and visualizations was performed in Python, utilizing an automated analysis pipeline^46^. In total, 285 and 4092 proteins were identified across all groups in ECF and CSF samples, respectively. For ECF, protein filtering for at least 70% of the group members was utilized in at least one group for pairwise comparisons. Only those samples containing at least 50% detected proteins of the median value (at least 45 proteins per sample) were used for the analysis. For CSF, to ensure high robustness, protein filtering for at least 90% of the group members in at least one group (including state and genotype) was utilized for pairwise comparisons. In both datasets, missing LFQ values were imputed by using a mixed model with downshift and KNN ^46^ (width= 0.3, standard deviation downshift= 1.8, knn_cutoff= 0.6). Identification of differentially expressed proteins between different brain states and genotypes was executed using t-test with permutation-based threshold FDR<0.05 at 250 randomizations. Sample correlations on the original datasets were calculated by the Pearson correlation coefficients. In extracellular fluid samples, coefficients of variation were between 48–58% and correlations between the groups comparable. In CSF, coefficients of variation between groups and correlations among biological replicates were comparable. The volcano plots were generated by applying FDR<0.05 and s0-value= 0.5, including 250 randomizations. Enrichment analysis (GOCC, GOMF, GOBP and Reactome) of significant proteins was performed with Fisher’s exact test and Benjamini-Hocberg FDR<0.05 cutoff. Venn plots were drawn with the Venny tool (https://bioinfogp.cnb.csic.es/tools/venny/index2.0.2.html), IPA protein location/type annotations with Quiagen software (https://digitalinsights.qiagen.com/), and the STRING annotations with an online tool (https://string-db.org/). The assigned molecular weights for extracellular fluid and CSF proteins were based on the native sizes of identified proteins, since sample preparation for mass spectrometry involves a protein digestion step.

### QUANTIFICATION AND STATISTICAL ANALYSIS

All statistical analyses, except for the proteomic dataset, were performed with GraphPad Prism (version 10.0, https://www.graphpad.com/). As a rule, the Kolmogorov-Smirnov test was conducted to assess the normality of the sample distribution before proceeding with analysis. For statistical analyses comparing two groups with normal distributions, we used an unpaired or paired t-test. Datasets including more than two groups were analyzed using one-way-ANOVA or two-way-ANOVA. For normally distributed datasets, Tukey’s or Sídák’s multiple comparisons test was used. If the sample did not pass normality test, the Mann-Whitney non-parametric test was used. The exact p values were calculated at a 0.05 level of significance. The significance levels were specified accordingly: *p < 0.05, **p < 0.01, ***p < 0.001, ****p < 0.0001. Specific statistical details can be found in the figure legends and method details section.

## Supplementary Material

1

2

3

4

5

Supplemental information can be found online at https://doi.org/10.1016/j.celrep.2024.114977.

## Figures and Tables

**Table:** KEY RESOURCES TABLE

REAGENT or RESOURCE	SOURCE	IDENTIFIER
Antibodies		
Anti-Iba1 antibody	Wako Chemicals	019-19741; PRID: AB_839504
Anti-Aqp4 antibody	Abcam	ab9512; PRID: AB_307299
Goat anti-Rabbit IgG (H+L) Highly Cross-Adsorbed Secondary Antibody, Alexa Fluor^™^ 488	Thermo Fischer Scientific	A-11034; PRID: AB_2576217
Goat anti-Mouse IgG (H+L) Cross-Adsorbed Secondary Antibody, Alexa Fluor^™^ 568	Thermo Fischer Scientific	A-11004; PRID: AB_2534072
Anti-vascular Amyloid 1-42 antibody	Abcam	ab201059; PRID: AB_2920640
anti-GAPDH antibody	Thermo Fischer Scientific	MA5-15738 RRID: AB_10977387
Goat anti-Mouse IgG (H+L) Secondary Antibody, HRP	Thermo Fischer Scientific	Catalog # 32430 RRID: AB_1185566
anti-GFP rabbit antibody	Donated by Ryohei Tomioka and Kathleen S. Rockland	N/A
Anti-β-Amyloid, 17-24 Antibody, 4G8	Covance	Catalog# SIG-39200
Bacterial and virus strains		
rAAV-hSyn-GRAB-NE2m(3.1)-WPRE-pA	BrainVTA	PT-2393
Chemicals, peptides, and recombinant proteins		
Alexa Fluor^™^ 488 Cadaverine	Thermo Fischer Scientific	A30676
Acetonitrile, MS grade	Merck	1000301000
2-Chloroacetamide	Merck	22790
Ethylene glycol	Thermo Fischer Scientific	29810
Glycerol	Thermo Fischer Scientific	J61059.K2
Congo Red	Sigma-Aldrich	# 101641
Neutral formalin buffer 10%	Hounisen	1000.1000
Isotonic saline solution	B. Braun Melsungen AG	02 93 10
Isopropanol, MS grade	Thermo Fisher Scientific	10091304
Lysyl EndopeptidaseR (Lys-C)	WAKO Chemicals	125-02543
Water, MS grade	VWR Chemicals	83645.290
Sodium deoxycholate	Merck	SRE0046
Trifluoroacetic acid	Merck	8082600101
Triton X-100	Thermo Fischer Scientific	85111
Tris(2-carboxyethyl)phosphine hydrochloride	Merck	C4706
Trizma base	Merck	T1503
Trypsin	Merck	T6567
Tween-20	Thermo Fischer Scientific	28360
Critical commercial assays		
V-PLEX Aβ Peptide Panel 1, 4G8	MSD	K15199E
Dynabeads^™^ Protein G for Immunoprecipitation	Thermo Fischer Scientific	10003D
Neurofilament Light Chain Assay	Quanterix	103186
Deposited data		
Matlab code for NE oscillation analysis	Github	https://github.com/MieAndersen/Sleep_NE_APP_PS1/tree/main
Proteomic datasets	PRIDE	PXD054763
Python code for proteomic analysis	Github	https://github.com/MannLabs/CKG
Experimental models: Organisms/strains		
B6;C3-Tg(APPswe,PSEN1dE9)85Dbo/Mmjax and non-transgenic (WT Ctrl) littermate mice on a C57BL/6;C3H background	University of Copenhagen	
Software and algorithms		
MATLAB	MathWorks	https://www.mathworks.com/products/matlab.html; RRID:SCR_001622
ImageJ software	NIH	https://imagej.net/ ; RRID:SCR_003070
GraphPad Prism 10	Dotmatics	https://www.graphpad.com/features; RRID: SCR_002798
Python 3.7.9	Python Software Foundation	https://www.python.org/ ; RRID:SCR_008394
EthoVision XT	Noldus	https://www.noldus.com/ethovision-xt; RRID:SCR_000441
LC solution	Shimadzu	https://www.shimadzu.com/
Venny online tool	BioTools	https://bioinfogp.cnb.csic.es/tools/venny/; RRID:SCR_016561
IPA	Quiagen	https://analysis.ingenuity.com/pa/installer/select ; RID:SCR_008653
STRING 12.0	STRING	https://string-db.org/; RRID:SCR_005223
Spectronaut 15.6	Biognosys	https://biognosys.com/software/spectronaut/
Other		
Mouse guide cannula 4.5 mm	Bilaney	C315GS
Mouse Dummy fits	Bilaney	C315DCS
Internal cannula fits	Bilaney	C315IS
1 mL syringe	Fisher Scientific	14955464
Blood collection tubes, EDTA	VWR	BDAM368841
Heraeus Multifuge X3R Centrifuge	Thermo Fisher Scientific	50121907-2
EvoSep One LC system	EvoSep Biosystems	EV-1000
Evotips	EvoSep Biosystems	EV-2000
Performance column	EvoSep Biosystems	EV1137
Lunatic spectrophotometer	Unchained Labs	47356
Microplate	Thermo Fischer Scientific	NP0341BOX
Microdialysis Probe, 7 kDa, 2 mm	Harvard Apparatus	CMAP000083
Microdialysis Probe, 500, 2mm	Harvard Apparatus	CMA8012422
NuPAGE^™^ Bis-Tris Mini Protein Gels	Thermo Fischer Scientific	NP0341BOX
NuPAGE MES SDS Buffer Kit	Thermo Fischer Scientific	NP0060
Pierce^™^ ECL western blotting substrate	Thermo Fischer Scientific	A43840
SDB-RPS material	Empore	66886-U
Silicone sealing mat	Axygen	AXYGAM-96-PCR-RD
Thermomixer C	Eppendorf	5382000015
TimsTOF Pro	Bruker	N/A
DAPI solution	Thermo Fischer Scientific	62248
Vacuum Concentrator	Eppendorf	5305000509

## Highlights

- Sleep deprivation increases the power of NREM norepinephrine oscillation in WT mice
- In contrast, sleep loss reduces the power of norepinephrine oscillations in APP/PS1 mice
- Amyloid-β protein aggregates in APP/PS1 mice 24 h after sleep deprivation
- The proteasomal pathway displays elevated upregulation upon sleep loss
