# Supplementary material for: Sleep deprivation leads to non-adaptive alterations in sleep microarchitecture and amyloid-β accumulation in a murine Alzheimer model
Source: Cell Rep. Author manuscript; Available in PMC 2025 Aug 26. (PMC12379227; doi:10.1016/j.celrep.2024.114977)
Supplement: 1 [file NIHMS2038914-supplement-1.pdf]

Cell Reports, Volume *43*

Supplemental information

Sleep deprivation leads to non-adaptive alterations in sleep microarchitecture and amyloid-β accumulation in a murine Alzheimer model

Neža Cankar, Natalie Beschorner, Anastasia Tsopanidou, Filippa L. Qvist, Ana R. Colaço, Mie Andersen, Celia Kjaerby, Christine Delle, Marius Lambert, Filip Mundt, Pia Weikop, Mathias Jucker, Matthias Mann, Niels Henning Skotte, and Maiken Nedergaard

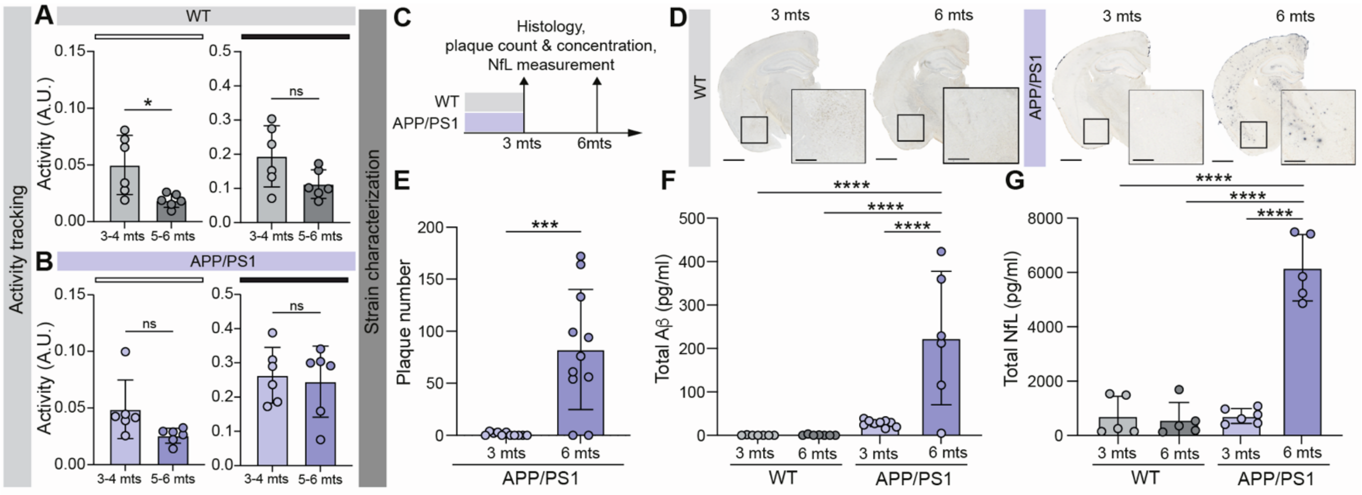

Figure S1. APP/PS1 mice show no age-related activity changes and no AD pathology at 3-months of age. Related to Figure 1.

(**A**) Total mouse activity in WT and (**B**) APP/PS1 genotype across inactive and active phases, WT p*= 0.0167, ns=0.07, APP/PS1 ns=0.06, ns=0.70, mean ± SD, unpaired-t-test, n=6. (**C**) Schematic diagram for mouse strain characterization. Histological plaque quantifications and ELISA measurements (brain Aβ and CSF Nfl) were performed at 3- and 6-months of age. (**D**) Histological representative Aβ-staining in coronal sections with the antibody 6E10 in APP/PS1 and WT animals. Scale bars represent 500 µm (slice) and 250 µm (inserts). (**E**) Number of plaques per each coronal slice in APP/PS1 animals at 3 and 6 months of age, mean ± SD, n=11, unpaired-t-test, ***p=0.0001, n=11. (**F**) Total brain concentration of Aβ (ELISA) in 3- and 6-months old WT or APP/PS1 animals, mean ± SD, n=6-9, One-way ANOVA with Tukey's multiple comparisons test, p****< 0.0001 (**G**) Total Nfl concentration in mouse CSF (ELISA), mean ± SD, n=5-6, One-way ANOVA with Tukey's multiple comparisons test, p****< 0.0001.

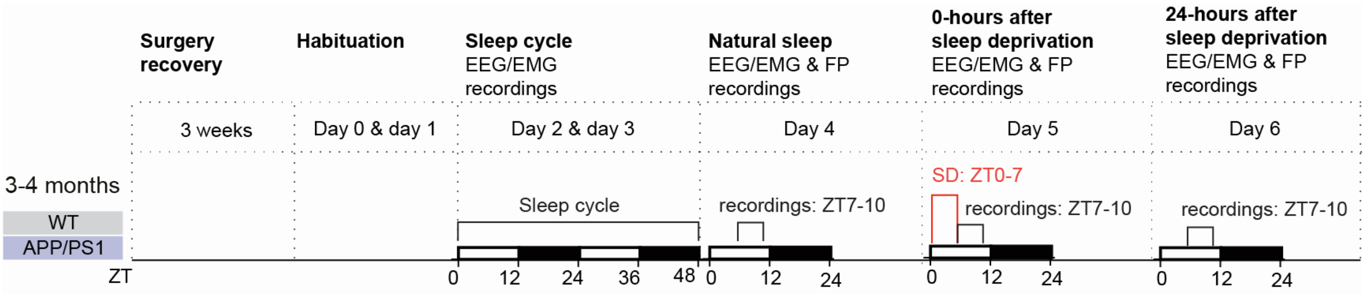

Figure S2. Experimental setup for EEG/EMG and FP. Related to Figure 1 and Figure 2.
Detailed scheme illustrating the experimental setup for sleep cycle EEG/EMG and 3-hour long EEG/EMG and FP recordings with corresponding zeitgeber times. SD: sleep deprivation, EEG/EMG: electroencephalogram/electromyography, FP: fiber photometry.

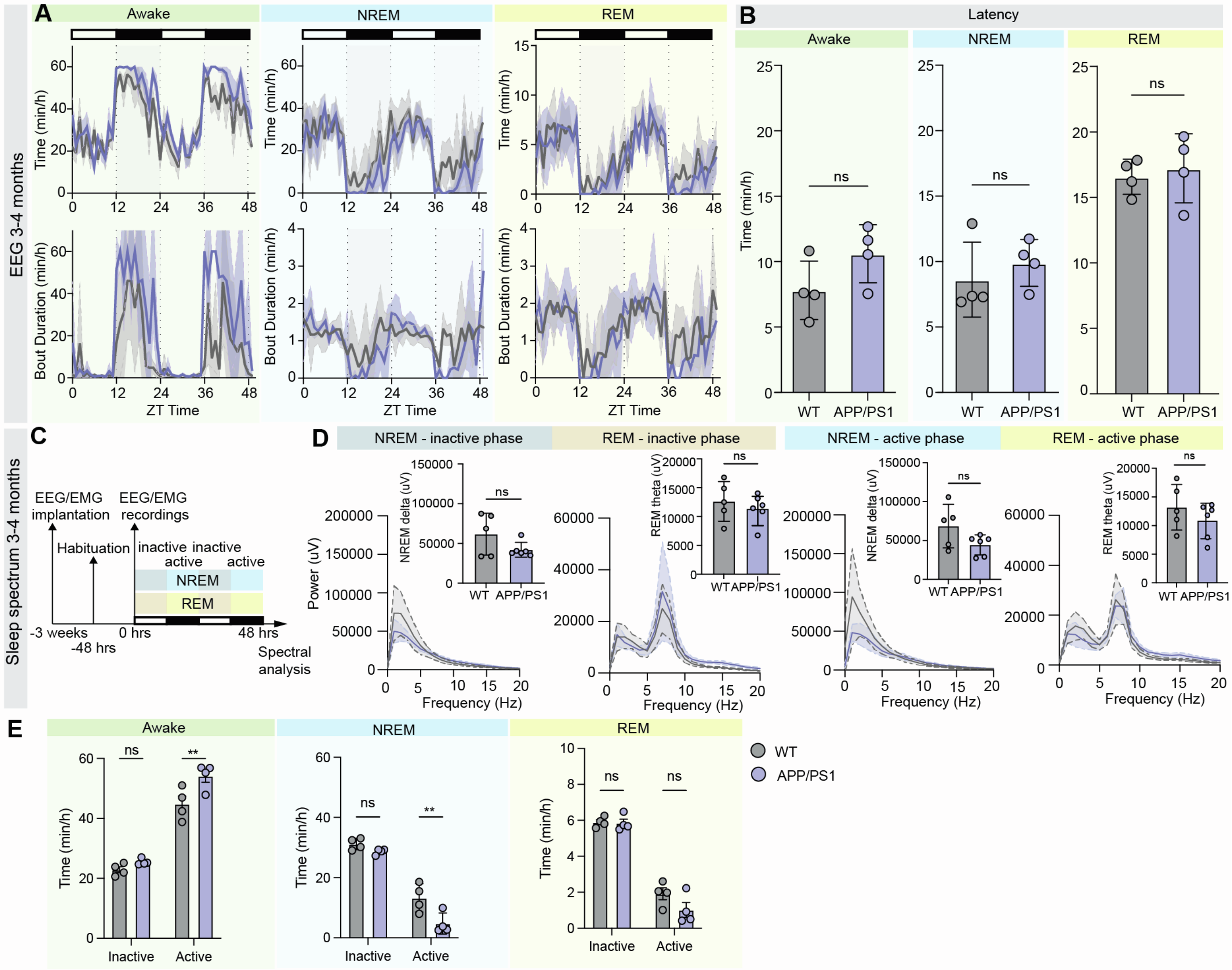

Figure S3. Young APP/PS1 mice show no changes in sleep latency or EEG spectrum. Related to Figure 1. (**A**) Percentage of time spent (left) awake, (middle) NREM, (right) REM, and (below) corresponding bout duration in each brain state, n=4. The EEG traces show ± SEM. (**B**) Histograms displaying latency for awake (left), NREM (middle) and REM (right) sleep. Unpaired t-test, values are average of two light/dark cycles, ns=0.128, ns=0.478, ns=0.677, n=4. (**C**) Schematic of EEG/EMG recordings. Averaged values from two inactive or active phase were used for spectral analysis. (**D**) (left) Spectral analysis of NREM and REM sleep during inactive phase. NREM: ns=0.12, REM: ns=0.5. (right) Spectral analysis of NREM and REM sleep during active cycle. NREM: ns=0.09, REM: ns=0.31. Spectrum traces show mean ± SD. Unpaired t-test, n=5-6. (**E**) Percentage of time spent (left) awake, (middle) NREM and (right) REM according to inactive or active phase. Two-way-ANOVA, awake ns=0.33, **p=0.003, NREM ns=0.27, **p=0.002, REM ns=0.90, ns=0.05, n=4. Bar graphs show mean ± SD.

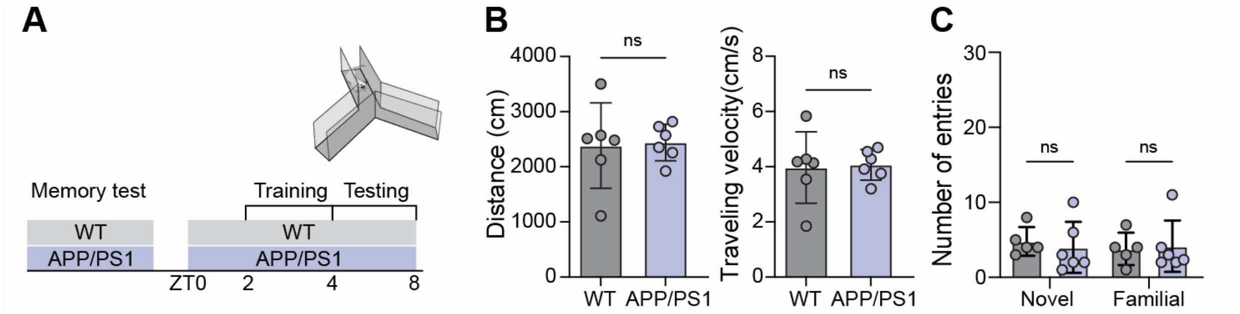

Figure S4. Spatial memory is not perturbed in young APP/PS1 mice. Related to Figure 1.
(**A**) Schematic of Y-maze behavioral experiment in baseline state during ZT4-8. (**B**) Quantification of moved distance and travelling velocity. Unpaired t-test, distance: ns= 0.869, velocity: ns=0.869, n=6. (**C**) Assessment of spatial working memory in baseline state, indicated by entry of familial or novel arm. One-way ANOVA, all ns, baseline: ns=0.879, ns=0.973, sleep deprivation: ns=0.679, ns=0.889, n=5-6. Bar graphs show mean ± SD.

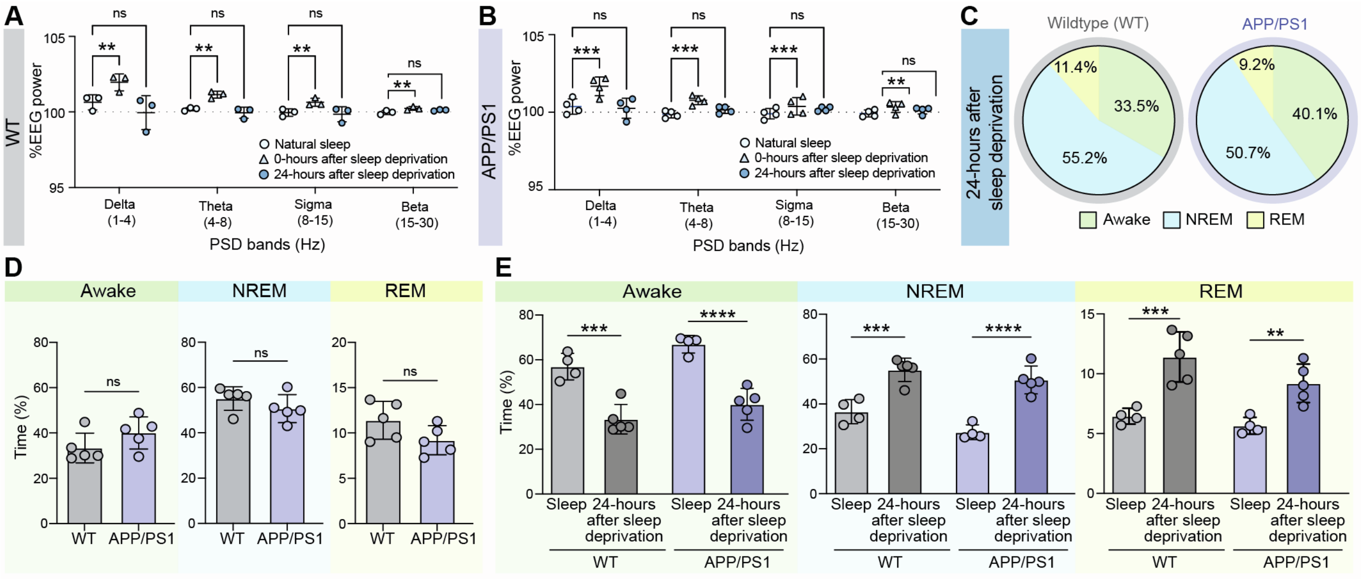

Figure S5. Young APP/PS1 mice show changes in EEG power in the first hour after sleep deprivation. Related to Figure 2.

(**A-B**) The %EEG power in (**A**) WT and (**B**) APP/PS1 mice during natural sleep, 0-hours after sleep deprivation or 24-hours after sleep deprivation at various frequency bands. Two-way ANOVA with Sidak's multiple comparisons test, WT: delta; ns=0.98, **p=0.006, theta; ns=0.98, **p=0.006, sigma; ns=0.98, **p=0.006, beta; ns=0.98, **p=0.006, APP/PS1: delta; ns=0.98, ***p=0.0001, theta; ns=0.98, ***p=0.0001, sigma; ns=0.98, ***p=0.0001, beta; ns=0.98, ***p=0.0001, n=3-4. Mean ± SD is shown. (**C**) Pie chart showing % time spent awake, in NREM 24-hours after sleep deprivation and REM 24-hours after sleep deprivation, in the 3-4 months group, n=5. (**D**) Total % time spent awake (left), in NREM 24-hours after sleep deprivation (middle) and REM 24-hours after sleep deprivation (right), all ns, n=5. (**E**) Comparison of total % time spent awake (left), in NREM (middle), and REM (right) phase during natural or 24-hours after sleep deprivation for each genotype, two-way ANOVA with Sidak's multiple comparisons test, awake: ***p=0.0001 and ****p<0.0001, NREM: ***p=0.0002 and ****p<0.0001, REM: ***p=0.0004 and **p=0.0061, n=4-5, Mean ± SD is shown.

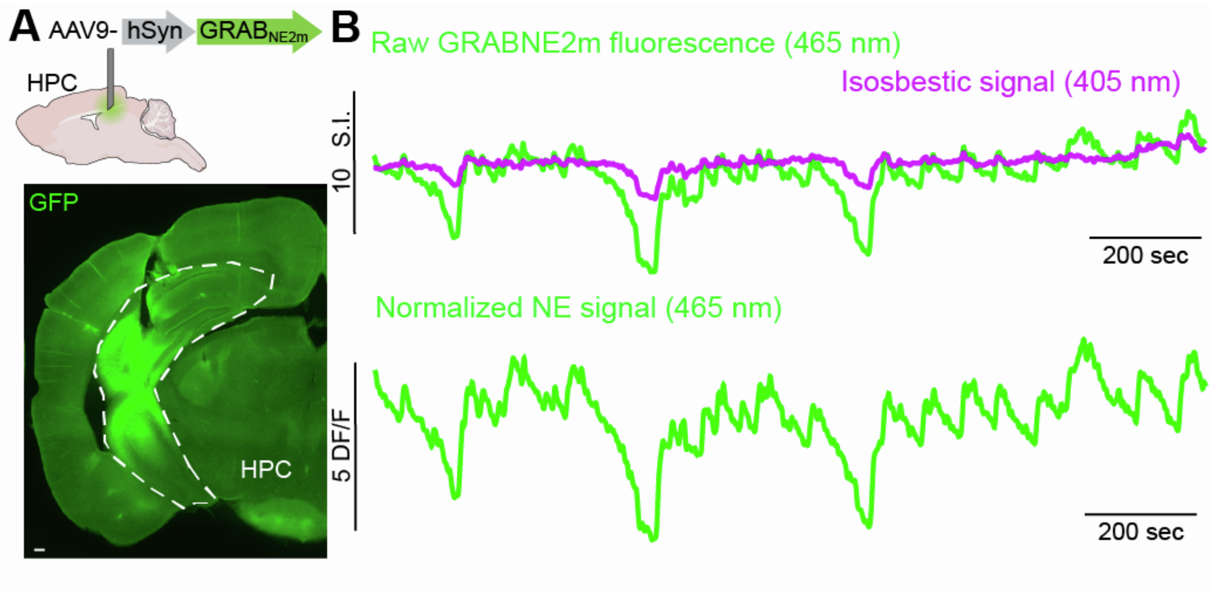

Figure S6. Assessment of viral expression and implantation of optical fiber. Related to STAR methods and Figure 2.

(**A**) Representative image of norepinephrine biosensor $GRAB_{NE2m}$ expression in hippocampus (HPC). Scale bar represents 500 µm. (**B**) Sample traces of raw $GRAB_{NE2m}$ and isosbestic control channel from one animal, filtered and down-sampled (above). NE signal after subtraction, filtered and down-sampled (below).

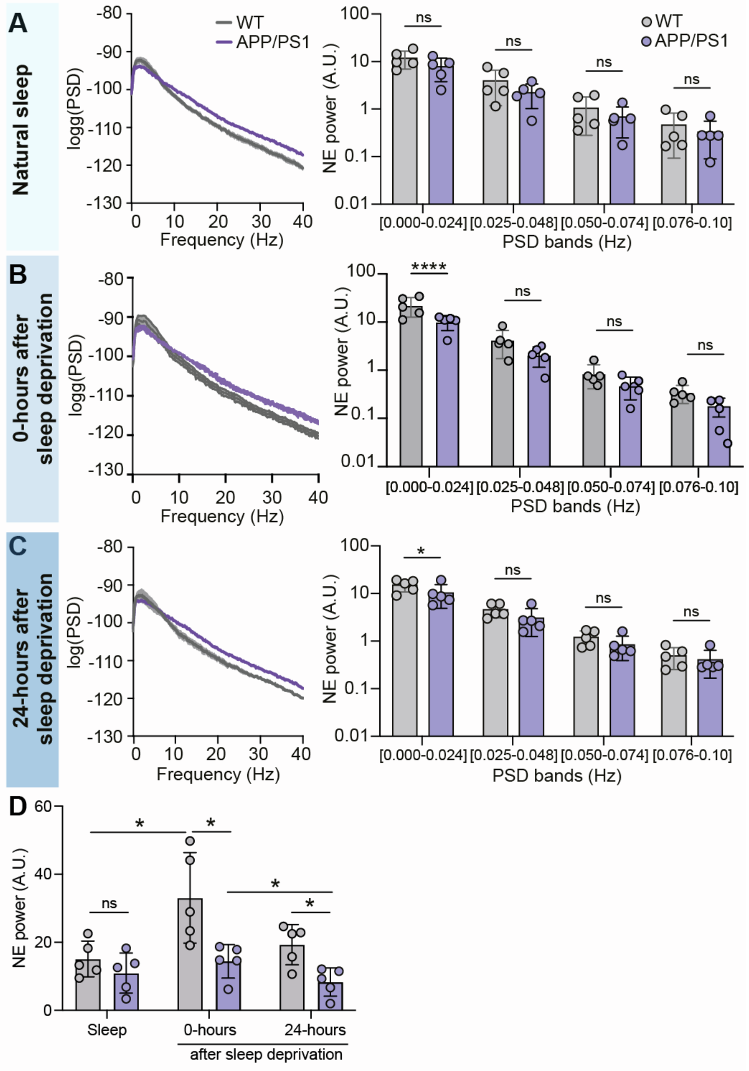

Figure S7. Mean power density is changed 0-hours and 24-hours after sleep deprivation in APP/PS1 mice. Related to Figure 2.

Power spectral density trace during natural NREM sleep (**A**), 0-hours after sleep deprivation (**B**) or 24-hours after sleep deprivation (**C**) and assessment of mean power densities across frequency bands in natural NREM (**A**) Natural NREM sleep, NREM sleep 0-hours after sleep deprivation (**B**) and NREM sleep 24-hours after sleep deprivation (**C**). During natural sleep all ns, 0-hours after sleep deprivation *p=0.02, and 24-hours after sleep deprivation ****p<0.0001, n=5, two-way ANOVA with Sidak's multiple comparison test. Bar graphs indicate mean ± SD. (**D**) Power spectra density analysis comparing NE power during natural sleep and 0-hours or 24-hours after sleep deprivation NREM sleep. WT vs APP/PS1: 0-hours after sleep deprivation *p=0.0318, 24-hours after sleep deprivation *p=0.0110, WT sleep vs 0-hours after sleep deprivation: *p=0.045, WT sleep vs 24-hours after sleep deprivation: ns=0.5, WT 0-hours after sleep deprivation vs 24-hours after sleep deprivation: ns=0.08, APP/PS1 sleep vs 0-hours after sleep deprivation: ns=0.4, APP/PS1 sleep vs 24-hours after sleep deprivation: ns=0.3, APP/PS1 0-hours after sleep deprivation vs 24-hours after sleep deprivation: *p=0.03. Paired t-test, n=5. Arbitrary units, A.U.

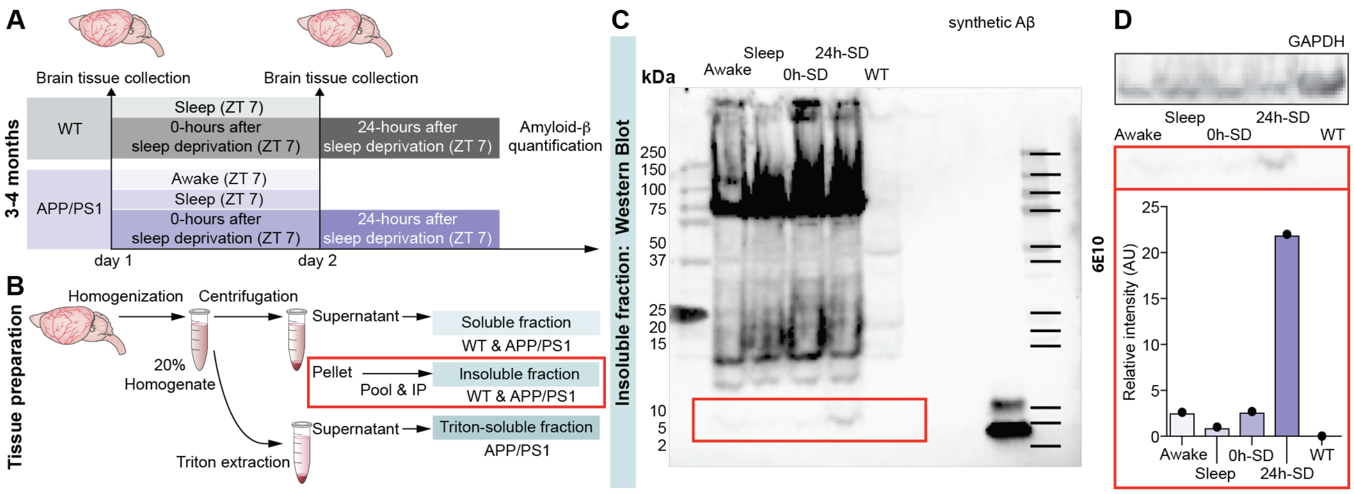

Figure S8. Elevation of insoluble Aβ 24-hours after sleep deprivation in APP/PS1 mice. Related to Figure 3. (**A**) Experimental setup for brain tissue collection. (**B**) Preparation of tissue samples for Western blot; insoluble (pellets pooled and immunoprecipitated) fractions. (**C**) Quantification of Aβ in the insoluble fraction detected with western blot (WB) using the 4G8 antibody, detecting human-specific Aβ overexpressed in APP/PS1 mice. Samples from six mice were pooled for each brain state, for one technical replicate. (**D**) The grey square portrays the GAPDH loading control for the samples, whereas purple square shows zoom in. Bar graph of the relative intensity (arbitrary units, AU) from a single WB.

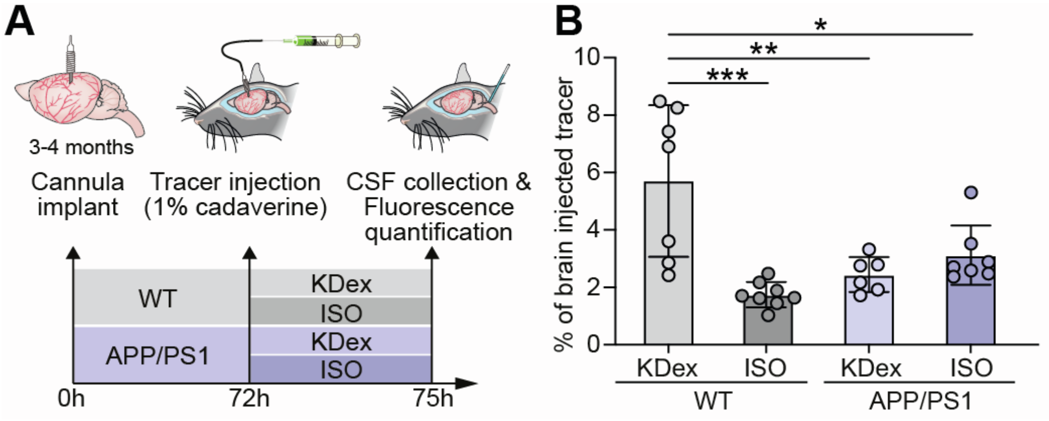

Figure S9. Glymphatic efflux of cadaverine is suppressed in young APP/PS1 mice. Related to Figure 3. (**A**) Schematic of glymphatic clearance experiment. (**B**) Percentage of hippocampally injected cadaverine tracer measured in CSF during KDEx or ISO anesthesia regimens in 3-4 months old WT and APP/PS1 mice. One-way ANOVA with Tukey's multiple comparisons test, *p=0.015, **p=0.0028, ***p=0.0001, n=6-8, bar graphs show mean ± SD. KDex: ketamine and dexmedetomidine, ISO: isoflurane.

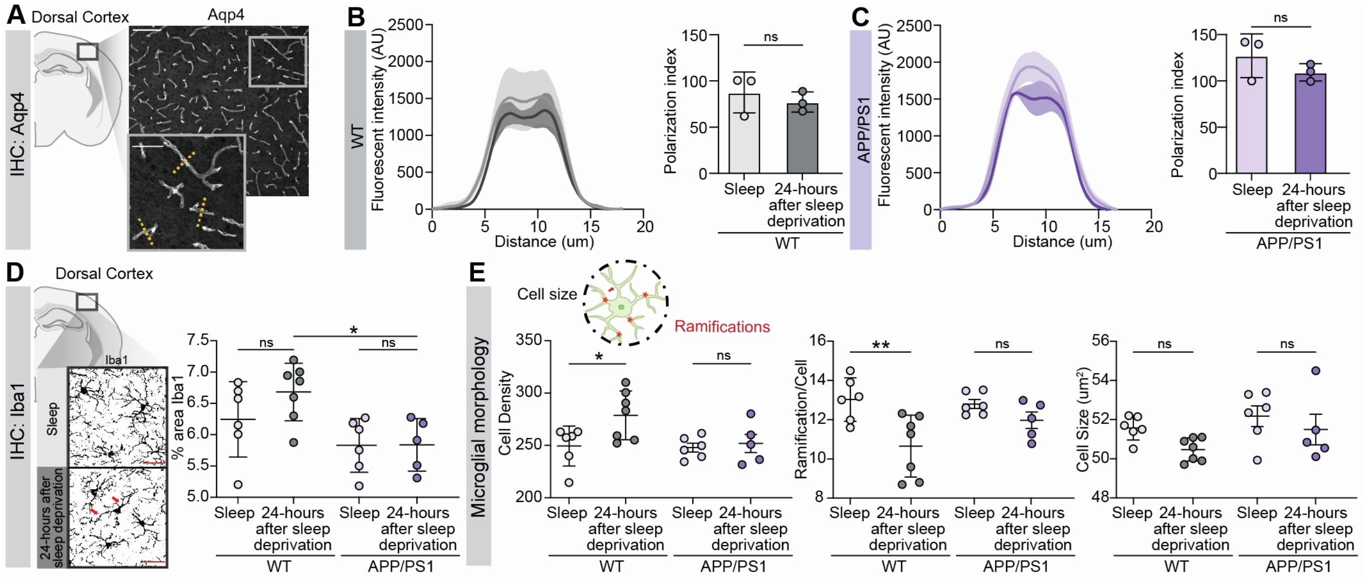

Figure S10. APP/PS1 mice show no loss of AQP4 polarization, but lesser microglial activation 24-hours after sleep deprivation Related to Figure 2 and Figure 3.

(**A**) Representative image of astrocytic marker AQP4. Orange dashed line represents example of line ROI traces that were plotted for polarization quantification. Scale bars represent 20 µm. (**B-C**). Left: Plots represent average fluorescent trace of 12–36 blood vessels per mouse (n=3). Summarized traces are shown as group averages ± SEM. Right: Polarization index (Arbitrary Units, A.U.) of AQP4 in (**B**) WT and (**C**) APP/PS1 mice in natural and 24-hours after sleep deprivation, Unpaired t-test, WT: ns=0.508, APP/PS1: ns=0.288, n=3. (**D**) Left: Schematic for Iba1 immunohistochemistry and representative staining in natural and 24-hours after sleep deprivation. Red arrow portrays changes in microglial ramifications. Red scale bars represent 20 µm. Right: Quantification of % total area stained with microglial marker. One-way ANOVA with Tukey's multiple comparisons test, *p =0.0348, n= 6-7. Plots show averaged values of 12 image fields from each mouse, n=5-6. (**E**) Left: Schematic for microglial morphology assessment. Dashed circle represents cell size and red asterisk show microglial ramifications. Right: Quantification of microglial morphological changes in natural and 24-hours after sleep deprivation in WT and APP/PS1 mice. One-way ANOVA with Tukey's multiple comparisons test, *p= 0.0228, **p= 0.0026, n=5-7. All graphs indicate mean ± SD.

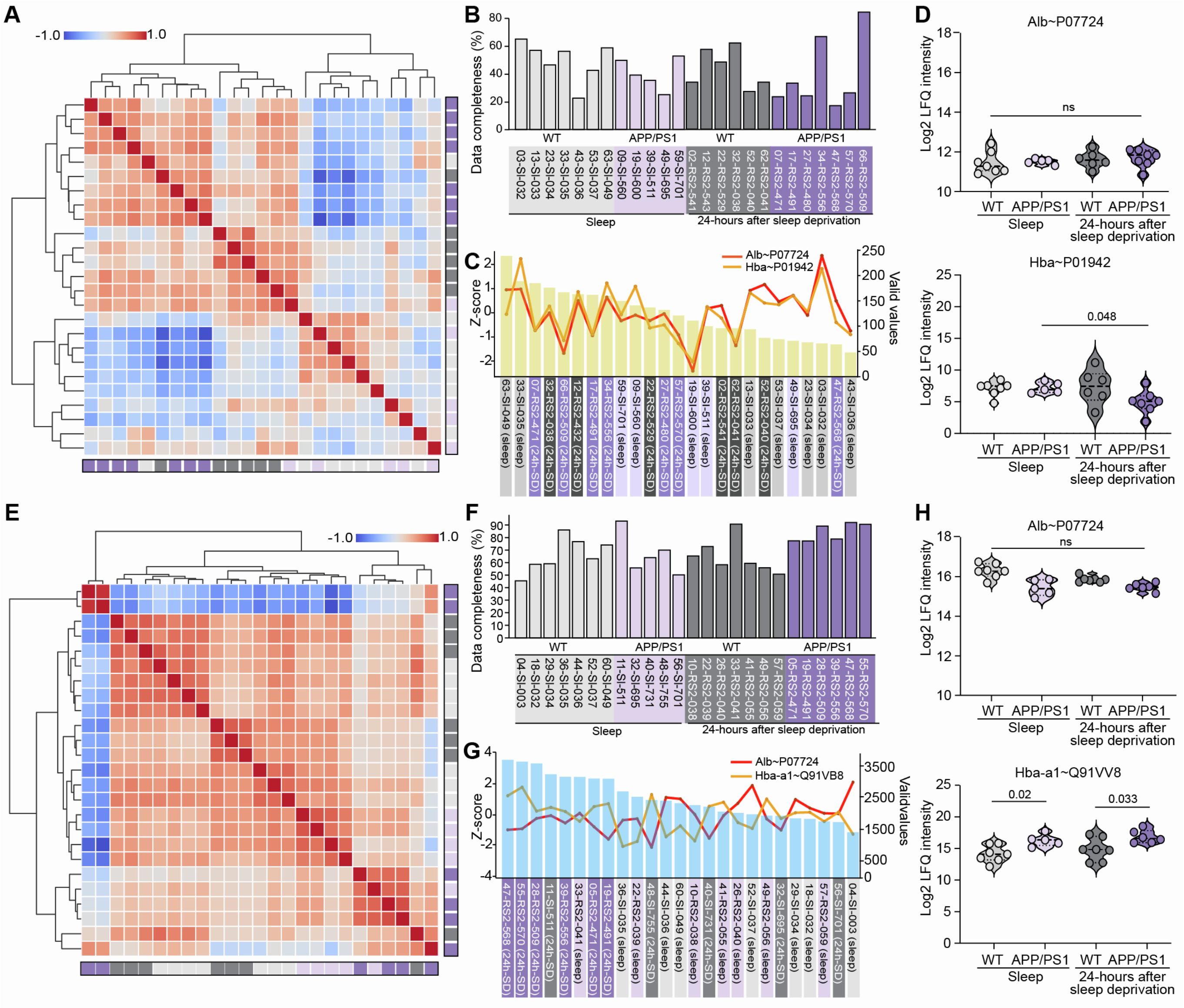

Figure S11. Quality control of extracellular fluid and CSF samples. Related to Figure 4 and Figure 5.
(**A**) Global sample correlation on imputed extracellular fluid dataset, generated by clustering the Pearson correlation coefficients for all sample combinations. Coefficient numbers are colour-coded. (**B**) Data completeness of each extracellular fluid sample before imputation, shown as percent (%). (**C**) Valid values across all samples and z-scored LFQ traces of albumin (red) and hemoglobin (orange) proteins. (**D**) Total protein levels of Alb and Hba-a1 proteins between genotypes, expressed as log (LFQ) label free quantification values. Permutation-based correction padj<0.05 values, n=5-7 mice. Violin plots show mean ± SD. (**E**) Global sample correlation on imputed CSF dataset, generated by clustering the Pearson correlation coefficients for all sample combinations. Coefficient number is colour-coded. (**F**) Data completeness of each CSF sample before imputation, shown as percent (%). (**G**) Valid values across all samples and z-scored LFQ traces of albumin (red) and hemoglobin (orange) proteins. (**H**) Total protein levels of Alb and Hba-a1 proteins between genotypes, expressed as (LFQ) label free quantification values. Permutation-based correction padj<0.05 values, n=5-7 mice. Violin plots show mean ± SD.

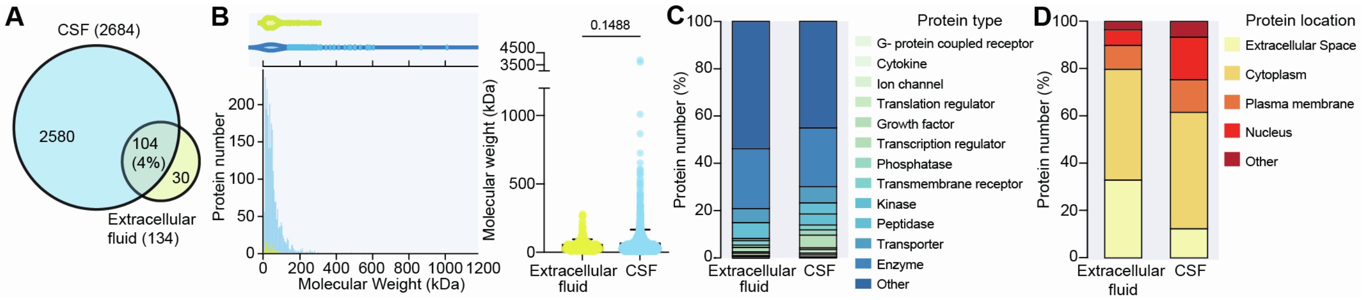

Figure S12. Proteome comparison of brain extracellular fluid and CSF. Related to Figure 4 and Figure 5.
(**A**) Venn diagram displaying all common and unique proteins between the extracellular fluid and CSF on imputed datasets. Total numbers of detected proteins are in brackets. (**B**) Histogram distribution of protein molecular weights in extracellular fluid and CSF for processed datasets (unpaired-nonparametric-t-test). (**C-D**) Stack plot illustrating (**C**) protein type and (**D**) cellular location of identified and IPA (Ingenuity Pathway Analysis) annotated proteins, expressed as percent (%). The IPA annotated 131 proteins in extracellular fluid 2,494 in CSF.

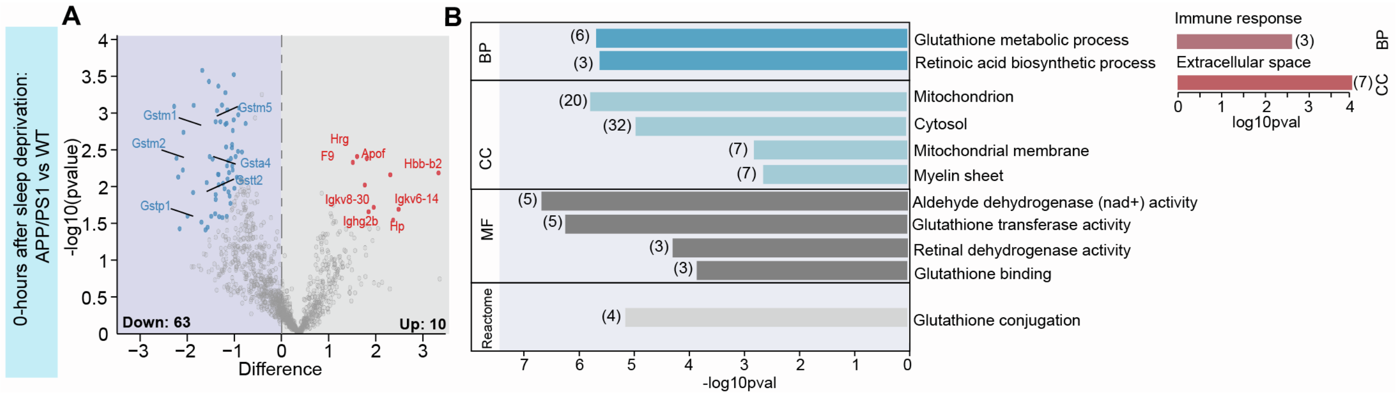

Figure S13. CSF proteome upon sleep deprivation indicates reduced antioxidant response in APP/PS1 mice. Related to Figure 5.

(**a**) Volcano plots representing protein changes 0-hours after sleep deprivation in APP/PS1 and WT group. Differential changes are indicated by red (upregulated) and blue (downregulated) dots, with p<0.05 (student's t-test with permutation correction) and two-fold change in log2. Gray dots are not significant, n= 5-7 mice per each genotype. Student's t-test with permutation correction, p<0.05. (**B**) Enrichment analysis of upregulated (red tones) and downregulated (blue tones) protein pathways after sleep deprivation, between APP/PS1 and WT. Number of proteins in each pathway is stated in the brackets.
