# Supplementary figures and images for: Sleep deprivation leads to non-adaptive alterations in sleep microarchitecture and amyloid-β accumulation in a murine Alzheimer model
Source: Cell Rep. Author manuscript; Available in PMC 2025 Aug 26. (PMC12379227; doi:10.1016/j.celrep.2024.114977)

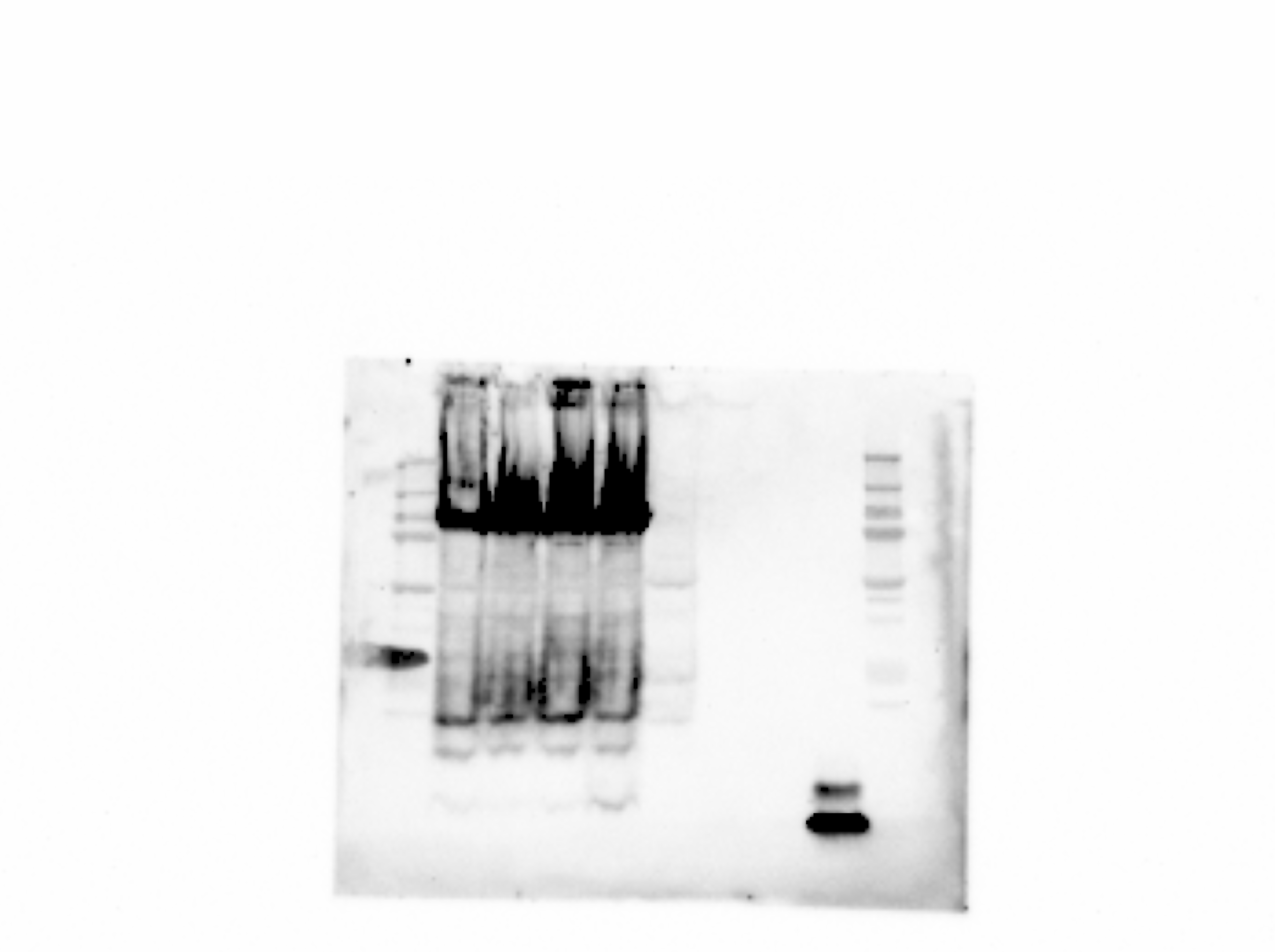

Supplement: 5 [file NIHMS2038914-supplement-5.zip › Unprocessed WB_pico_Moc31_IP_SNfraction_180s_NC.tif]
